# A new basal sauropod from the pre-Toarcian Jurassic of South Africa: evidence of niche-partitioning at the sauropodomorph–sauropod boundary?

**DOI:** 10.1038/srep13224

**Published:** 2015-08-19

**Authors:** Blair W. McPhee, Matthew F. Bonnan, Adam M. Yates, Johann Neveling, Jonah N. Choiniere

**Affiliations:** 1 Evolutionary Studies Institute, University of the Witwatersrand, Private Bag 3, Johannesburg, Gauteng, 2050, South Africa; 2 DST/NRF Centre of Excellence for Palaeosciences, University of the Witwatersrand, Private Bag 3, Johannesburg, Gauteng, 2050, South Africa; 3 School of Geosciences, University of the Witwatersrand, Private Bag 3, Johannesburg, Gauteng, 2050, South Africa; 4 Biology Program, Stockton University, 08205-9441, Galloway, New Jersey, United Sates of America; 5 Museum of Central Australia, Araluen Cultural Precinct, P.O. Box 3521, Alice Springs, Northern Territory, 0871, Australia; 6 Council for Geoscience, Pretoria, 0001, South Africa

## Abstract

The early evolution of sauropod dinosaurs remains poorly understood, with a paucity of unequivocal sauropod taxa known from the first twenty million years of the Jurassic. Recently, the Early Jurassic of South Africa has yielded an assemblage of dental and post-cranial remains displaying a more apomorphic character suite than any other similarly aged sauropodomorph. These remains are interpreted as a new species of basal sauropod and recovered cladistically as the sister taxon to *Vulcanodon* +more derived Sauropoda, underscoring its importance for our understanding of this pivotal period of sauropod evolution. Key changes in the dentition, axial skeleton and forelimb of this new species suggest a genuine functional distinction occurring at the sauropodiform-sauropod boundary. With reference to these changes, we propose a scenario in which interdependent refinements of the locomotory and feeding apparatus occurred in tandem with, or were effected by, restrictions in the amount of vertical forage initially available to the earliest sauropods. The hypothesized instance of niche-partitioning between basal sauropodan taxa and higher-browsing non-sauropodan sauropodomorphs may partially explain the rarity of true sauropods in the basal rocks of the Jurassic, while having the added corollary of couching the origins of Sauropoda in terms of an ecologically delimited ‘event’.

Sauropod dinosaurs are justly famous for their redoubtable size, long geological reign, and unique physiology. However, prior to the early Middle Jurassic, relatively little is known of the early period of sauropod evolution. Although a number of non-sauropodan sauropodomorph taxa are known from the first twenty million years of the Jurassic^1^,^2^,^3^,^4^,^5^,^6^,^7^, only a handful of similarly aged taxa have been described as basal sauropods - an assignation that remains equivocal for most, if not all. *Chinshakiangosaurus* from the Fengjiahe Formation of China is based on a single dentary that displays an ambiguous mixture of primitive and derived characters^8^, while other Lower Jurassic Chinese basal ‘sauropods’ await either formal description (‘*Kunmingosaurus*’), or are too fragmentary to diagnose (‘*Zizhongosaurus*’)^9^,^10^. *Lamplughsaura* from the Upper Dharmaram Formation of India is of uncertain phylogenetic affinity, being recovered as either a stem taxon close to the base of Sauropodomorpha, or as a basal sauropod close to ‘*Kotasaurus’* and *Vulcanodon* within the one cladistical assessment of its relationships^11^. The latter taxon, long considered the earliest-known exemplar of the basal sauropod condition^12^, has been temporally reallocated to a sedimentary lens contained within the early Toarcian Drakensburg Group basalts^13^,^14^, while the putative Early Jurassic age of the Indian Kota Formation (which, along with ‘*Kotasaurus’*, also contains the basal sauropod *Barapasaurus*) is poorly supported, with both the dinosaurian and mammalian faunal assemblage suggestive of a late Early Jurassic age at the oldest^15^. Furthermore, Triassic sauropods such as *Antetonitrus* and *Isanosaurus* have been recently found to either be poor analogs for the basal sauropod condition^16^, or incorrectly dated^17^.

Here we describe material belonging to a new medium-sized sauropodiform dinosaur possessing a more apomorphic character suite than anything previously collected from within the Early Jurassic upper Elliot Formation of South Africa. This material contributes not only to our understanding of the timing of the genesis and subsequent radiation of Sauropoda, but also helps elucidate macroevolutionary and palaeoecological trends pertaining to the guild-structuring and functional diversity of Sauropodomorpha within the earliest Jurassic.

Institutional abbreviations: BP: Evolutionary Studies Institute (previously Bernard Price Institute), University of the Witwatersrand, South Africa.

## Results

SYSTEMATIC PALAEONTOLOGY

Saurischia Seeley 1888

Sauropodomorpha von Huene 1932

Sauropodiformes Sereno 2007 (sensu^16^)

Sauropoda Marsh 1878

*Pulanesaura eocollum* gen. et sp. nov.

### Holotype

The neural arch of an anterior dorsal vertebra (BP/1/6882) that is missing the dorsal apex of the neural spine.

### Type locality and horizon

**Figure 1:**
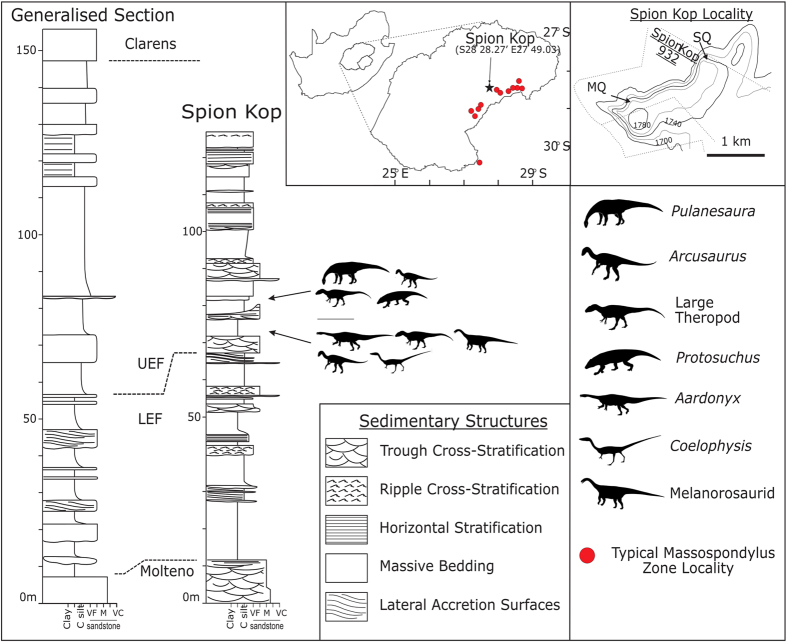
Stratigraphic succession of Spion Kop Farm, illustrating the faunal assemblages recovered from the upper Elliot Formation as preserved on the farm. SQ, ‘Sauropod [*Pulanesaura*] Quarry’; MQ, ‘Marc’s [*Aardonyx*] Quarry’. All cartographic information was recorded by JN and reproduced using Inkscape (vers. 0.91). All dinosaur silhouettes were drawn by the authors except for the one next to *Arcusaurus*, which is licensed under the CC-BY-SA GNU Free Documentation License and is attributed to Arthur Weasley. The original image and use policy for the image is available here: http://en.wikipedia.org/wiki/File:Thecodontosaurus.jpg.

The *Pulanesaura* material was obtained from a small (3 m × 3.5 m) quarry on the farm Spion Kop 932 in the Senekal District of the Free State, South Africa (Fig. 1). The quarry is located just over a kilometer East-North East of the holotype locality of *Aardonyx celestae*, in a higher stratigraphic position than that taxon within the early Jurassic upper Elliot Formation. The much smaller *Arcusaurus pereirabalorum* was recovered from the edge of the same quarry, and a detailed schematic of the excavation is figured in^18^ (see also Supplementary Information Fig. S1). The upper Elliot Formation on Spion Kop consists of a series of stacked channel sandstone bodies with little intervening overbank siltstones, the quarry itself being situated in a poorly bedded, coarse to sandy siltstone lens. The two-dimensional geometry and internal facies relationships of this lens suggests that it represents the fill of a low-energy, cut-off channel. Most age estimates suggest that the upper Elliot Formation is no younger than the Pliensbachian (183–191mya), with a consensus range of late Hettangian to Sinemurian (i.e., ~200mya or younger^19^,^20^).

### Referred material

The Spion Kop assemblage is composed of the partial remains of at least two subadult-to-adult individuals. The referred material is considered conspecific with the holotype with respect to the following: a) the bones were found in close association in a fine matrix with no evidence of high velocity transport; b) the different bones give a consistent phylogenetic signal that argues against a random aggregation of taxa having been brought together from a wide area in a flowing fluvial regime; and c) duplicated elements show no evidence of character conflict or other factors that may suggest the presence of more than one species of large-bodied, derived sauropododiform dinosaur. The referred material consists of: 2 teeth; mid-cervical vertebra; five dorsal neural arches; a single right dorsal rib; three caudal vertebra; left clavicle; distal right humerus; left ulna; ?right fourth metacarpal; three ischia; left and right tibiae; two first pedal unguals (see Supplementary Information for catalogue details). The humerus was recovered from a lens within the main quarry but approximately 1m below the rest of the material. While this element is provisionally referred to *Pulanesaura* with reference to the above criteria, its relative disassociation from the rest of the material means it is excluded from the diagnosis.

### Etymology

“*Pulane*”, Sesotho, meaning “rain-maker/bringer”, in reference to the rain-soaked conditions under which the dinosaur was excavated, plus ”-saura”, Latin, feminine, meaning “lizard”; “eo”, Greek, meaning “dawn”, plus “collum”, Latin, meaning “neck”, in reference to the hypothesized function of the neck presaging the sauropod condition in the new taxon.

### Diagnosis

A medium-sized transitional sauropodiform dinosaur, the holotype (BP/1/6882) of which is diagnosed with respect to a unique set of characters (autapomorphies marked with an asterisk): Neural spine high and anteriorly inclined; prezygapophyses mediolaterally extensive and sheet-like*; and anterior infradiapophyseal fossae showing an externally constricted, medially-tapering, ‘pinched’ morphology*.

The referred material is diagnosed thusly: Teeth with apicobasal grooves on both the labial and lingual surfaces, denticles restricted to the apical third of the crown, and crowns with extensive enamel wrinkling easily discernible with the naked eye; anterior-to-middle cervical vertebrae with anteroposteriorly short and dorsoventrally high neural spines and dorsally-raised, obliquely-set postzygapophyseal articular facets; dorsoventrally tall neural spines in the anterior dorsal vertebrae, being approximately three times taller than anteroposteriorly long; middle-to-posterior dorsal neural arches with neural spines over 1.6 times as high as long; anterior caudal vertebra with incipient prezygadiapophyseal laminae; anterior caudal transverse processes laterally restricted, triangular in shape and located on both the neural arch and centrum, the latter being almost twice as high dorsoventrally as anteroposteriorly long and lacking a ventral sulcus; hyposphene on anterior caudal vertebra; mediolaterally expansive radial fossa on the proximal ulna; tibia with a proximal surface over twice as long anteroposteriorly than transversely wide with similarly transversely restricted shaft; transversely compressed first pedal ungual with a convexly rounded proximoventral margin.

### Description

**Figure 2:**
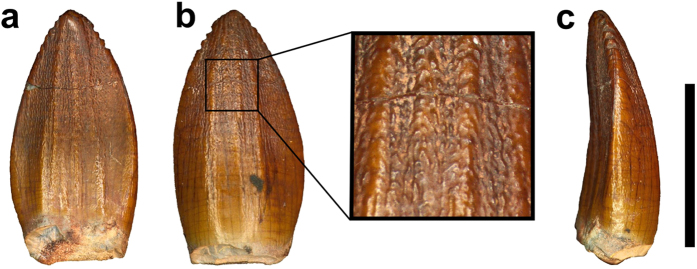
Tooth of *Pulanesaura eocollum* (BP/1/6204) in (**a**) lingual view; (**b**) labial view with expanded detail of tooth surface; (**c**) ?mesial view. Scale bar equals 1cm. Photographs by BWM.

The crowns of two isolated teeth, both of which are broken at the root-crown juncture, are known for *Pulanesaura*. Their semi-spatulate shape is similar to that of many non-sauropodan sauropodomorphs and most basal sauropods (e.g., *Tazoudasaurus*^21^, *Barapasaurus*^22^, and *Shunosaurus*^23^) (Fig. 2). Both teeth are ‘D’-shaped in cross-section with a strongly convex labial surface, while the larger of the two (BP/1/6204) is slightly lingually recurved. Denticles are present on the apical third of the crown but are only minimally expressed. Despite being both phylogenetically and serially plastic, denticles restricted to the apical third are present in most basal sauropods (e.g., *Spinopohorosaurus*^24^, *Barapasaurus*^22^,^25^). The teeth display strong apicobasal fluting on both the labial and lingual surfaces, with this being especially pronounced on the latter surface in which a series of grooves radiate symmetrically from either side of the mesiodistal midline. These grooves are interpreted as incipient lingual sculpting, which is present in all eusauropods^26^. Prominent enamel wrinkling is present in the apical half of the tooth crown, as in sauropods^25^.

**Figure 3:**
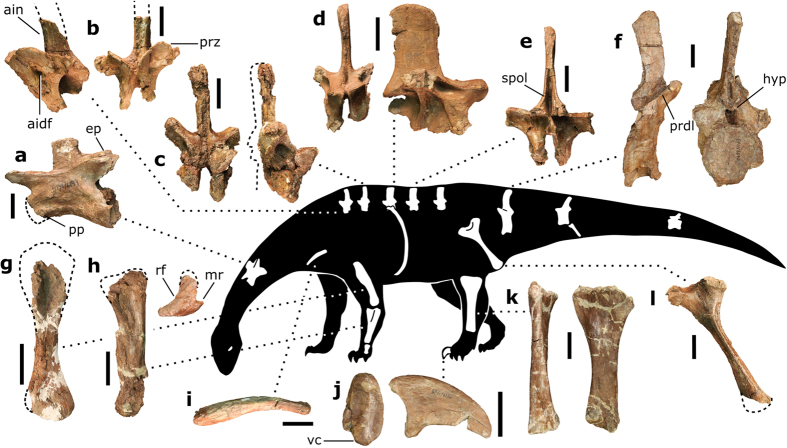
Representative bones of *Pulanesaura eocollum*. (**a**) anterior-to-middle cervical vertebrae (BP/1/6199) in left lateral view; (**b**) holotypic anterior-most dorsal neural arch (BP/1/6882) in left lateral and anterior views; (**c**) anterior dorsal neural arch (BP/1/6984) in anterior and right lateral views; (**d**) anterior mid-dorsal neural arch (BP/1/6183) in anterior and right lateral views; (**e**) middle dorsal neural arch (BP/1/6770) in posterior view; (**f**) anterior caudal vertebra (BP/1/6646) in right lateral and posterior views; (**g**) right humerus (BP/1/6193) in anterior view; (**h**) left ulna (BP/1/6210) in lateral and proximal views; (**i**) ?left clavicle (BP/1/6752) in dorsal view; (**j**) left pedal ungual I (BP/1/6186) in proximal and medial views; (**k**) left tibia (BP/1/6200) in anterior and lateral views; (**l**) right ischium (reversed) (BP/1/7366) in lateral view. Abbreviations: aidf, anterior infradiapophyseal fossa; ain, anterior incline of the neural spine; ep, epipophysis; hyp, hyposphene; mr, medial ridge; pp, parapophysis; prdl, prezygodiapophyseal lamina; prz, prezygapophyses; rf, radial fossa; spol, spinopostzygapophyseal lamina; vc, ventral convexity. Scale bars equal 5 cm in a-f and i, j; 10 cm in g, h, k, l. Silhouette drawn by BWM Photographs by BWM.

**Figure 4:**
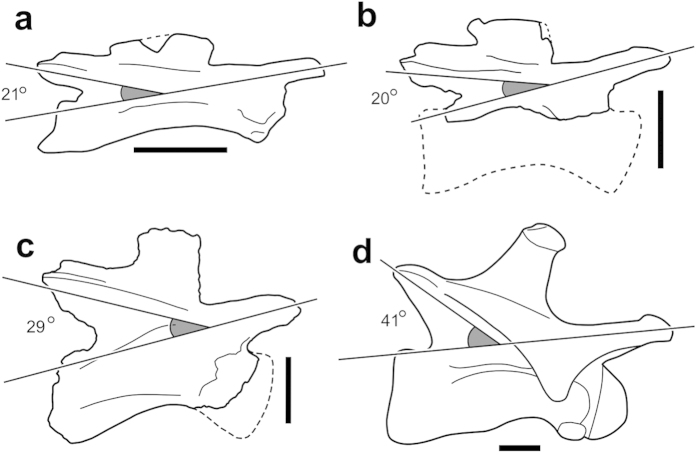
Changes in morphology of the anterior-to-middle cervical vertebrae throughout Sauropodomorpha. (**A**) *Massospondylus* (BP/1/5241); (**B**) *Aardonyx* (BP/1/6662); (**C**) *Pulanesaura*, (BP/1/6199); (**D**) *Patagosaurus* (from^29^). Scale bars equal 5cm.

The single preserved cervical vertebra is probably from the anterior to middle part of the neck and is missing the anterior end of the centrum, precluding determination of whether the bone was opisthocoelous as in more derived sauropods or amphicoelous as in all known non-sauropodan sauropodomorphs^2^ (Fig. 3a). The centrum is acamerate as in non-sauropodan sauropodomorphs, but is lower than the neural arch in the dorsoventral height of the posterior face, a derived feature shared with all sauropods (sensu^27^). The diapophyses are preserved as low tubercles, consistent with the interpretation of the vertebra as from the anterior half of the cervical series, while the absence of pronounced diapophyseal laminae retains the plesiomorphic condition for Sauropodomorpha (although see^4^). However, the postzygapophyses are elevated with respect to the prezygapophyses, with the former dorsally offset from the sagittal plane by about 30°, a morphology consistently observed in basal sauropods (e.g., ‘*Kotasaurus*’^28^, *Tazoudasaurus*^21^, *Patagosaurus*^29^, Fig. 4). The neural spine is comparatively tall for an anterior/middle cervical, with its maximum dorsoventral height roughly equivalent to its anteroposterior length.

There are five dorsal neural arches preserved, representing each region of the dorsal axial column (Fig. 3b–e). With the exception of the features of the holotypic dorsal vertebra mentioned in the diagnosis (see also Fig. S2), the most remarkable aspect of the dorsal vertebrae of *Pulanesaura* is the great relative height of the neural spines of the anterior dorsal vertebrae, which are approximately three times as high as anteroposteriorly long (Fig. 3c). In progressively posterior dorsal vertebrae the neural spines increase in length along the sagittal axis, changing from a distinctly anteroposteriorly compressed morphology in the anterior-most elements to more mediolaterally narrow, anteroposteriorly elongate neural spines from the mid-dorsals onwards (Fig. 3d). The neural spines of the middle-to-posterior dorsals nonetheless remain relatively high for basal Sauropodomorpha, with their dorsoventral height being over 1.6 times the length of their respective bases. Spinal laminae are restricted to the sheet-like spinopostzygapophyseal laminae (especially prevalent in the mid-dorsals onwards) that form large, buttressing structures between the postzygapophyses and the posterior margins of the neural spine (Fig. 3e). These structures are finer, and the post-spinal recess that they frame deeper, than the same processes in *Antetonitrus* (BP/1/4952).

Pneumatic sculpting is possibly present within the posterior infradiapophyseal fossa of one of the posterior dorsal neural arches, a morphology described in detail in Yates *et al*.^30^.

The centrum of the anterior caudal vertebra is biconcave, with its posterior facet almost twice as high as the anteroposterior length of the ventral surface, lending a considerably anteroposteriorly compressed morphology to the bone (Fig. 3f). This morphology is only known in *Tazoudasaurus*^21^ outside of Eusauropoda. There is a pronounced offset between the anterior and posterior articular facets, with the ventral margin of the posterior articular facet located at a level ventral to that of the anterior facet. No sulcus is present on the ventral surface of the centrum. The neural spine is three times higher than anteroposterior length of the base, proportionally taller than in any other taxon known from the Elliot Formation. Although the dorsal margins of both transverse processes/diapophyses are not preserved, the well-preserved struts of bone that extend ventrolaterally from the prezygapophyses strongly suggest the presence of low yet well-defined prezygadiapophyseal laminae, possibly representing the incipient development of the laminar configuration seen in the anterior caudals of more derived sauropod taxa (e.g., *Tazoudasaurus; Mamenchisaurus*^31^). The anteroposteriorly short transverse processes are preserved as laterally abbreviated, wedge-shaped protuberances than span the neurocentral juncture, showcasing the derived condition within Sauropodomorpha. Ventral to the postzygapophyses a small yet well-developed hyposphene is present.

The single ?left clavicle is spatula-shaped, with a tapered medial end and an expanded lateral end (Fig. 3i). The element is broadly triangular in cross-section, with the apex of the triangle directed dorsally, similar to clavicles of *Massospondylus* (BP/1/5241). The ventral surface of the expanded lateral end is heavily striated, suggesting strong ligamentous attachments with the acromial region of the scapula. The clavicle is moderately bow-shaped in dorsal view, with the dorsoventrally flattened lateral end directed posteriorly with respect to the medial end. This contrasts with the comparatively straight clavicles of more derived sauropod dinosaurs (e.g., *Spinophorosaurus*^24^, *Omeisaurus*^32^; *Jobaria*^33^).

Although the proximal end of the humerus is very poorly preserved, the general morphology is reminiscent of that of *Vulcanodon*. This is most evident with respect to the long, anteroposteriorly narrow humeral shaft that extends proximally to over half the preserved length of the humerus, the minimal transverse expansion of the distal condyles, and the absence of a clearly defined cuboid fossa^12^ (Fig. 3g).

The ulna is missing the proximal articular surface (Fig. 3h). Nonetheless, a deep, mediolaterally extensive radial fossa is readily observable^34^. The medial process of the proximal ulna of *Pulanesaura* is distinct in being a well-defined strut of bone that rises from about the mid-height of the shaft, appearing to become increasingly anteroposteriorly narrow towards its apex. This differs from the condition observed in *Antetonitrus* in which the medial process is an obtusely delineated ridge of bone that is thicker anteroposteriorly than laterally (BP/1/4952). However, it is possible that this feature is due to deformation.

The manus is currently represented by a single metacarpal IV, which is triangular in proximal view and stout in general proportions, consistent with the semi-stout, possibly load-resisting morphology of the manus of derived sauropodiforms.

The ischium retains the plesiomorphic sauropodomorph condition of a long, cross-sectionally triangular ischial shaft with a dorsoventrally expanded distal end (Fig. 3l).

The tibia showcases the ‘vulcanodontid’ condition of having a proximal articular surface is over twice as long anteroposteriorly than transversely wide (Fig. 3k). As in *Antetonitrus* and more derived sauropodiforms, this surface is relatively flat with respect to the horizontal plane, lacking the anterodorsal incline of the proximal end seen in more basal forms. Nonetheless, the cnemial crest of *Pulanesaura* retains the basal condition for Sauropodomorpha insofar as the anterior-most projection of the crest also represents the highest proximal point of the tibia. In the basal sauropods *Vulcanodon*^12^*, Tazoudasaurus*^21^ and *Spinopohorosaurus*^24^ the anterior-most projection of the cnemial crest is located approximately at the proximodistal midpoint of the process. The shaft of the tibia is mediolaterally compressed in a similar, if slightly less pronounced, manner to that of *Tazoudasaurus* and *Vulcanodon*. In contrast, non-sauropodan sauropodomorphs tend to display tibial shafts that are subcircular in cross-section.

Similar to the condition in basal sauropods, pedal ungual I is a tall, mediolaterally compressed bone with a ventrally convex proximal surface (Fig. 3j). In contrast, the first pedal ungual of derived non-sauropodan sauropodiform taxa (e.g., *Antetonitrus*; *Blikanasaurus*) tends towards a dorsoventrally squat morphology in which the proximal surface is ventrally flat^16^.

**Figure 5:**
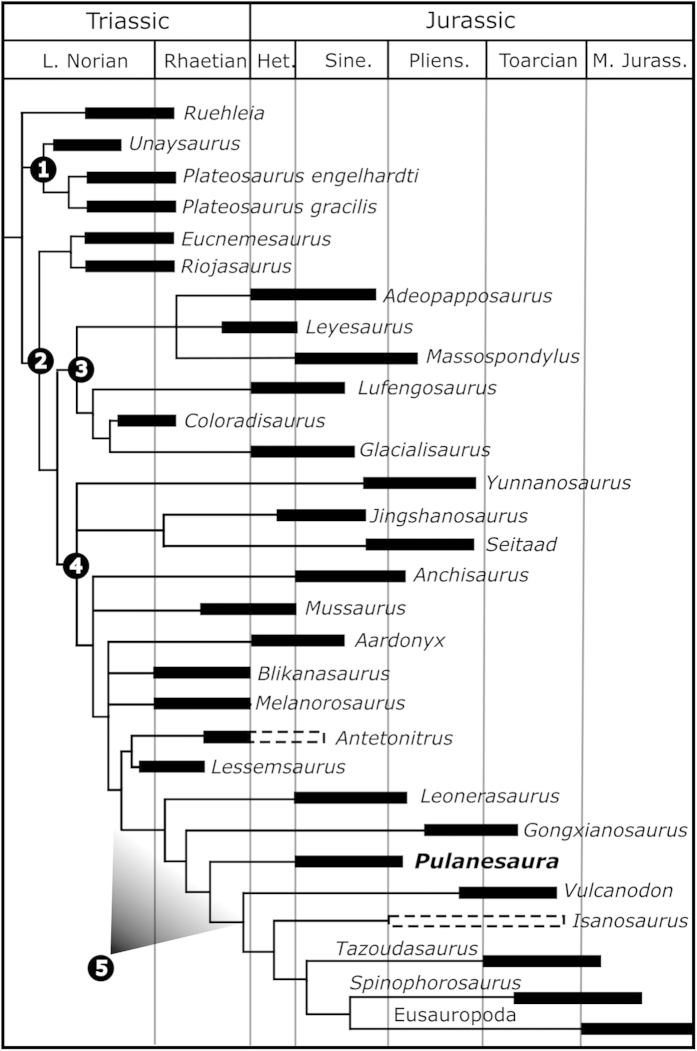
Abbreviated strict consensus tree showing relationships and hypothesised stratigraphic ranges of plateosaurian dinosaurs (*sensu*^27^). Dashed lines represent uncertainty in temporal duration. 1, Plateosauridae; 2, Massopoda; 3, Massospondylidae; 4, Sauropodiformes; 5, Sauropoda (node left undesignated in order to reflect current disagreements regarding the taxonomic definition of Sauropoda [see text]).

A cladistic analysis of the relationships of *Pulanesaura* was conducted using a modified version of the data matrix of McPhee *et al*.^35^, resulting in 69 MPTs with a shortest length of 1264 (see Supplementary Information). The strict consensus tree of these MPT’s resolves *Pulanesaura* as the sister-taxon to *Vulcanodon* + more derived sauropods (Fig. 5). This position is supported by the following unambiguous synapomorphies: pneumatic sculpting within the posterior infradiapophyseal fossa of the dorsal vertebrae (ch. 162); height of mid-dorsal neural spines greater than 1.5 times the length of the base (due to a possible reversal to the plesiomorphic state in *Gongxianosaurus*) (ch. 167); well-developed, sheet-like spinopostzygapophyseal laminae in the dorsal vertebrae (ch. 171); anterior caudal transverse process extending from the neural arch to the centrum (ch. 188); and a dorsoventrally tall, transversely flattened first pedal ungual (ch. 367). The derived position of *Pulanesaura* is also supported by a number of characters that are either rendered ambiguous due to a lack of information at nodes immediately basal or apical to it, or present a slightly more inclusive distribution (see Supplementary Information). These include: coarsely wrinkled tooth enamel (ch. 117); longitudinal grooves on the labio-lingual surfaces of the teeth (ch. 119); mid-cervical neural arches higher than the posterior face of the centrum (ch. 133); hyposphenal ridge on the anterior caudal vertebra (ch. 186); prezygadiapophyseal laminae on the anterior caudal vertebra (ch. 187); absence of a well-defined flexor fossa on the anterior surface of the distal humerus (ch. 213); proximal surface of the tibia over twice as long anteroposteriorly than transversely wide (ch. 310); and the anteromedial corner of the distal tibia forming a non-acute, right angle (ch. 315).

Because of differing taxonomic opinions on the node-or-stem-based definition for Sauropoda, our phylogenetic hypothesis of *Pulanesaura* places it as either a basal sauropod (*sensu*^27^,^36^, or as the sister taxon to Sauropoda (*sensu*^16^,^37^,^38^). Regardless of taxonomic definition, *Pulanesaura* is the most derived sauropodiform known from the Elliot Formation or securely aged contemporaneous deposits worldwide for which its phylogenetic relationships can be stated with relative confidence.

## Discussion

*Pulanesaura* is part of an increasingly taxonomically diverse group of sauropodomorph dinosaurs from the upper Elliot Formation. Although the exact age of these deposits is still under investigation^39^, it is clear from multitaxon deposits at localities like Spion Kop^4^,^18^ that at least some Elliot sauropodomorphs lived contemporaneously (Fig. 1), suggesting the presence of guild-level divisions amongst sympatrically associated taxa. Although niche partitioning via differential feeding strategies and neck mechanics of contemporaneous Eusauropoda (i.e., ‘low-browsing’ diplodocoids vs. ‘high-browsing’ titanosauriforms) has been discussed at length in the literature (e.g.,^40^,^41^,^42^,^43^), it has never been proposed as an explanatory model for the diversity of basal sauropodomorph taxa in Late Triassic/Early Jurassic deposits like the Elliot Formation. Although the exploitation of increasingly larger vertical foraging ranges is often cited as a key ecological driver in the origins of the sauropodan bauplan (e.g.,^44^,^45^,^46^), this fails to explain the continuing numerical superiority of non-sauropodan sauropodomorphs for most of the Early Jurassic, many of which were of comparable size to the earliest sauropods (e.g., *Jingshanosaurus*; *Aardonyx*).

The derived suite of features present in *Pulanesaura* place it as sister to Sauropoda (or as a basal member of this taxon depending on phylogenetic definition) and strongly differentiates it from other known Elliot sauropodomorphs. These features relate primarily to changes in the feeding apparatus (wrinkled enamel in the dentition), axial morphology (non-planar cervical zygapophyseal facets; high neural spines in both cervical and anterior dorsal vertebrae) and forelimb (lack of flexor-pit on the distal humerus; deep radial fossa on the proximal ulna). These features, and their departure from the plesiomorphic sauropodomorph condition, indicate a genuine functional distinction occurring at the very base of Sauropoda with implications for our understanding of both the basal foraging strategy, and evolutionary context, of the sauropodan condition.

Most sauropodomorph taxa known from the latest Triassic through the earliest Jurassic, including those thought to be antecedent to the sauropodan bauplan (e.g., *Melanorosaurus; Lessemsaurus*; *Antetonitrus*) retain the semi-abducted, flexed forelimb posture plesiomorphic to Sauropodomorpha^47^. In some taxa, e.g., *Antetonitrus*, osteological markers suggest the presence of hypertrophied caudofemoralis brevis musculature that would have assisted with occasional bipedal locomotion^16^. The combination of a mobile grasping hand, flexed forelimb, and at least some degree of facultative bipedality probably represents an early solution to a catholic feeding regime in the first bulk-browsing dinosaurian herbivores. While rearing, and with the neck extended and raised at an oblique angle to the substrate, some of the larger non-sauropodan sauropodiform taxa could have fed at heights of ~5 meters. With the majority of the available forage thought to have occurred between ground level and 6 meters^44^, this would have enabled exploitation of the great majority of the browsing gallery.

However, while a dexterous hand and flexed/abducted forelimb would have been of appreciable utility in either grasping at foliage and/or supporting the body while leaning sub-vertically against the trunks of large trees, these same features may have compromised sauropodomorph fitness at some point along the size continuum^48^,^49^. For example, in modern eutherian mammals, all taxa with masses exceeding 300 kg have erect, non-crouched forelimbs to best mediate the increased compressive stresses on the bones^50^. Nonetheless, although the fossil trackway record suggests that facultative-habitual quadrupedality appears early in sauropodomorph evolution (e.g.,^51^) (potentially as a response to the increasingly high intake of low-quality vegetable matter and the large gut-capacities required to process it), the majority of evidence suggests a substantial delay between this novel locomotor strategy and meaningful alterations in the biomechanical efficiency of the forelimb^16^. In this respect, the uniquely massive scapular blade of non-sauropodan sauropodiform taxa such as *Yunnanosaurus*, *Lessemsaurus*, and *Antetonitrus* (and possibly *Gongxianosaurus*)^5^,^16^,^52^ possibly represents a trade-off between the additional anchorage required to counteract the increased shear stresses experienced by a large-bodied quadruped with a less than erect forelimb on the one hand, and the necessary mobility of the forelimb for bipedal high-browsing on the other. Likewise, the autapomorphically long cervical vertebrae of the smaller, possibly habitually bipedal Massospondylidae can also be viewed in the context of a trade-off between overall body-size and the need to feed across as wide a range of the trophic sphere as possible^53^.

In contrast to the above, the appendicular and axial morphology showcased by *Pulanesaura* potentially relates to a concerted change in the postural and behavioural locomotor complex towards an energetically conservative form of specialised low-to-mid browsing at the base of Sauropoda. In basal non-sauropodan taxa (e.g., *Plateosaurus*; *Massospondylus*; *Aardonyx*) the line of articulation across the zygopophyses of all non-posterior cervical vertebrae is only minimally offset from the sagittal. In *Pulanesaura* and other basal sauropods (e.g., ?*Lamplughsaura; Tazoudasaurus*; *Shunosaurus*; *Patagosaurus*), this relationship alters dramatically, with the postzygapophyses being offset from the prezygapophyses by as much as 40° (Fig. 4). It is possible that this change reflects alterations in the kinematic potential of the sauropodomorph neck, especially with respect to degrees of flexion along the dorsoventral axis. Similarly, the apomorphically tall anterior dorsal neural spines of *Pulanesaura* (and other basal sauropods) may instance the reorganisation and/or hypertrophy of the posterior epaxial neck musculature as a means of counteracting tensile stresses while the neck is held at a low-to-horizontal angle, while also affording additional purchase for the large dorsal neck ligaments responsible for storing the elastic energy instrumental in recovery from a ventrally flexed position^54^. The expansive, sheet-like prezygapophyses of the anterior-most dorsal vertebra also bear mention as potential bracing mechanisms at the base of the neck.

These changes, when considered alongside aspects of the *Pulanesaura* forelimb that indicate a more erect, columnar stance (reduction in flexor anatomy, anteriorly braced proximal radius), suggest the development of simultaneous and possibly interdependent innovations towards a non-grasping, fully parasagittal forelimb in concert with a neck with more anterior flexibility via a posteriorization of its muscle architecture^55^. While the coarse enamel wrinkling characteristic of sauropod teeth is of unknown functional significance, the possibility that it is related to differing functional requirements for processing flora commonly encountered at the low-to-mid browsing ranges (possibly juvenile and/or small members of the ‘seed-fern’ and pinophytan groups^56^) warrants future investigation. Taken in aggregate, this suite of features is strongly suggestive of a feeding strategy concentrated upon the lower ranges of the total available forage, differentiating *Pulanesaura* from contemporaneous sauropodomorphs that engaged in high-browsing, at least occasionally. Furthermore, the presence of similar (if less developed) character suites in other ‘near-sauropod’ forms such as *Leonerasuarus*^57^ and *Lamplughsaura*^11^ is suggestive of a foraging strategy that potentially optimizes as an ancestral condition for Sauropoda itself.

Recently, Sander^58^ and Sander *et al*.^45^, have modelled a series of evolutionary cascades, each dependent on a constellation of both primitive and novel influences, which led to the unique gigantism of sauropod dinosaurs. Many of these influences pivot upon physiological traits that are either plesiomorphic (e.g., lack of tooth-on-tooth occlusion [i.e., mastication], long neck, small head) or derived (e.g., invasive post-cranial pneumaticity) for Sauropodomorpha. While the exaptive potential of these traits in facilitating the high body masses of sauropod dinosaurs has been convincingly demonstrated in recent years^45^, the timing and coalescence of these traits in terms of the diversification and global dispersal of Sauropoda is still poorly known^25^,^59^.

The additional information provided by *Pulanesaura* places alterations of the neck and forelimb at the base of a potentially novel cascade feature in which the temporary abandonment of the higher reaches of the browsing gallery led to the breaking of locomotory constraints inhibiting true gigantism. Sander^58^ places “upright stance” at the base of the cascade “metabolism”, citing the obvious energetic advantages of a limb that is oriented in the manner of an inverted pendulum, while the long neck of sauropod dinosaurs is interpreted as being ecologically and adaptively advantageous at a number of cascade levels. However, while ‘upright stance’ is mentioned only with reference to the hindlimb within the expanded model^58^, both Remes^47^ and Sander *et al*.^45^, draw attention to the shift from an adductor-driven to an abductor-driven locomotory system in the forelimb paralleling the move towards gigantism in the early evolution of Sauropoda. Unfortunately, at our current anatomical resolution, changes in axial structure appear at the same time as changes in forelimb structure^29^, so it is unclear whether modifications of the neck drove postural changes or vice versa. Furthermore, the lack of preservation of pivotal anatomical structures in *Pulanesaura* (e.g., scapula, complete manus/pes), as well as the persistent incompleteness of the early sauropod record, continues to obscure a clear reading of whether the shift to a fully erect forelimb occurred in a stepwise fashion from near-sauropod grade animals such as *Antetonitrus*, or represents a genuine breakaway bioecological strategy of Sauropoda *sensu stricto*.

Nonetheless, we emphasize that the adoption of a less laterally oriented forelimb, as facilitated by the anteroventral rotation of the glenoid (thus bringing the forelimb more directly in line with the vector of the ground reaction forces) and a deepening of the radial fossa of the ulna, in tandem with specific changes in the architecture of the anterior axial column, represent perhaps the most important, non-pneumatic contributions to the cascade of traits ultimately leading to sauropod gigantism^34^,^47^. Contextually, these changes convey an instance of niche partitioning amongst contemporaneous sauropodomorph taxa within the earliest Jurassic Elliot Formation of South Africa, one in which the loss of a regularly assumed bipedal posture placed increasing selective and mechanical pressure upon the neck to become the critical food-gathering organ, requiring in turn a stable, erect base with which to support this organ. Although obligate quadrupedality has been inferred as early as the Late Triassic with respect to the relatively derived *Isanosaurus*^60^, the temporal provenance of this taxon should be treated with caution in light of recent doubts regarding the temporal relationships of the Nam Phong Formation^17^.

While the scenario outlined above is hypothesised to have played an important role in taking basal sauropods out of direct competition with sympatric non-sauropodan sauropodomorph dinosaurs capable of efficient rearing, it nonetheless would have limited the amount of vegetable matter accessible to them to within only a few meters from the ground^46^, potentially explaining the rarity of sauropod dinosaurs within the earliest Jurassic ecosystems. As previously noted by McPhee *et al*.^16^, the major burst of sauropod diversification seems to have only occurred towards the end of the Early Jurassic, suggesting an initial lag between the suite of locomotory and biomechanical novelties already present in basal taxa like *Pulanesaura*, and the inferred spike in sauropodan fitness ultimately conferred by these novelties (see also^21^).

The unique suite of characters unequivocally shared between *Pulanesaura* and taxa immediately apical to it further supports a divergence time for Sauropoda *sensu stricto* close to the Triassic-Jurassic boundary. While rare constituents of the sauropodomorph faunal assemblage, the discovery of *Pulanesaura* demonstrates that early sauropods were nonetheless active upon the desiccated floodplain of the upper Elliot Formation, buoying the prospects for other mid-to-low-latitude early Jurassic formations to also yield basal sauropod remains. Additional finds and increased anatomical and taxonomic resolution is required in order to better test the model presented here, and to further disentangle the ecological dynamics at play in the diversification and respective specialization of different groups of sauropodomorph dinosaurs. Isotopic analysis of dental remains as a means of assessing the reality of differential trophic-level interactions between various browsing strategists would assist in the exploration of this latter question. However, biomechanical and calorific considerations are likely to prove the crucial factors in future investigations of the energetic advantages and disadvantageous inherent in the diverse modes of locomotion and food acquisition that the sauropodomorph-sauropod transition is only just now beginning to reveal.

### Methods and Materials

Excavation and preparation: Exposed *in-situ* bone was consolidated using a dilute solution of Paraloid B-72 in acetone solvent. Once consolidated, the specimens were excavated with the use of both a rock saw and hand tools including rock hammers, chisels, and shovels. They were removed from the ground in plaster jackets composed of layers of burlap and plaster of Paris. During this process they were protected by a layer of newspaper dampened in water. Rock matrix was removed from the specimen in the lab primarily with handheld pneumatic airscribes. Fossilized bone was consolidated using an approximately 10% solution of Paraloid B-72 solid grade thermoplastic acrylic resin in 100% acetone solvent. Individual pieces of bone were glued together using either cyanoacrylate (various brands) or a highly concentrated (~30%) solution of Paraloid B-72 in 100% acetone solvent.

The phylogenetic analysis of *Pulanesaura* was drawn from the data matrix originally introduced by Yates^27^ and subsequently employed (with various alterations) by a number of other sauropodomorph workers (e.g.,^1^,^16^,^35^,^36^). The data matrix (see supplementary material), comprising 55 taxa and 365 characters, was analysed using TNT 1.1^61^ using a heuristic search of 1000 replicates of Wagner trees followed by TBR branch swapping with 10 trees saved per replication. Characters were equally weighted. The following 40 multistate characters were treated as ordered: 8, 13, 19, 23, 40, 57, 69, 92, 102, 117, 121, 131, 144, 147, 149, 150, 157, 162, 167, 170, 177, 205, 207, 225, 230, 237, 245, 254, 257, 270, 283, 304, 310, 318, 338, 351, 354, 356, 361, 365.

## Additional Information

**How to cite this article**: McPhee, B. W. *et al*. A new basal sauropod from the pre-Toarcian Jurassic of South Africa: evidence of niche-partitioning at the sauropodomorph–sauropod boundary? *Sci. Rep*. **5**, 13224; doi: 10.1038/srep13224 (2015).

## Supplementary Material

Supplementary Information
