# Supplementary material for: A new basal sauropod from the pre-Toarcian Jurassic of South Africa: evidence of niche-partitioning at the sauropodomorph–sauropod boundary?
Source: Sci Rep. 2015 Aug 19;5:13224. doi: 10.1038/srep13224 (PMC4541066; doi:10.1038/srep13224)
Supplement: Supplementary Information [file srep13224-s1.pdf]

A new basal sauropod from the pre-Toarcian Jurassic of South Africa: evidence of niche-partitioning at the sauropodomorph–sauropod boundary?

BLAIR W. MCPHEE, MATTHEW F. BONNAN, ADAM M. YATES, JOHANN NEVELING, and JONAH N. CHOINIERE.

SUPPLEMENTARY INFORMATION

Contents:

1) Catalogue information for *Pulanesaura* material from Spion Kop quarry
2) Figure S1: *Pulanesaura* quarry map
3) Figure S2: Additional images of holotype
4) Figure S3: Example of *in situ* fossils
5) Table S1: Measurements
6) Modifications to previous matrix
7) Extended results (explanation of ambiguous synapomorphies)
8) Character list
9) Character-by-taxon matrix

1) List of remains

Tooth, complete minus root (BP/1/6204); tooth, missing root and tip of crown (BP/1/6207); anterior-to-middle cervical vertebra (BP/1/6199); anterior dorsal neural arch from cervicodorsal transition (BP/1/6882: HOLOTYPE); poorly preserved anterior dorsal neural arch (BP/1/6984); anterior middle dorsal neural arch (BP/1/6183); middle dorsal neural arch (BP/1/6770); posterior dorsal neural arch (BP/1/6183a); anterior caudal vertebra (BP/1/6646); mid-anterior caudal vertebra (BP/1/6201); mid-to-distal caudal vertebra (BP/1/7741); dorsal rib (BP/1/6768); chevron (BP/1/6205); clavicle (BP/1/6752); right humerus (BP/1/6193); left ulna (BP/1/6210); metacarpal IV (BP/1/6191); mostly complete right ischium (BP/1/7366); proximal portion of left ischium (BP/1/6184); smaller right ischium (BP/1/6202); left tibia (BP/1/6200); right tibia (BP/1/6980); complete first pedal ungual (BP/1/6186); proximal portion of pedal ungual (BP/1/6983).

Note: The non-sequential cataloguing pattern of the *Pulanesaura* material reflects disparate preparation times between elements.

2) Figure S1: Map of the Sauropod Quarry from Spion Kop Farm

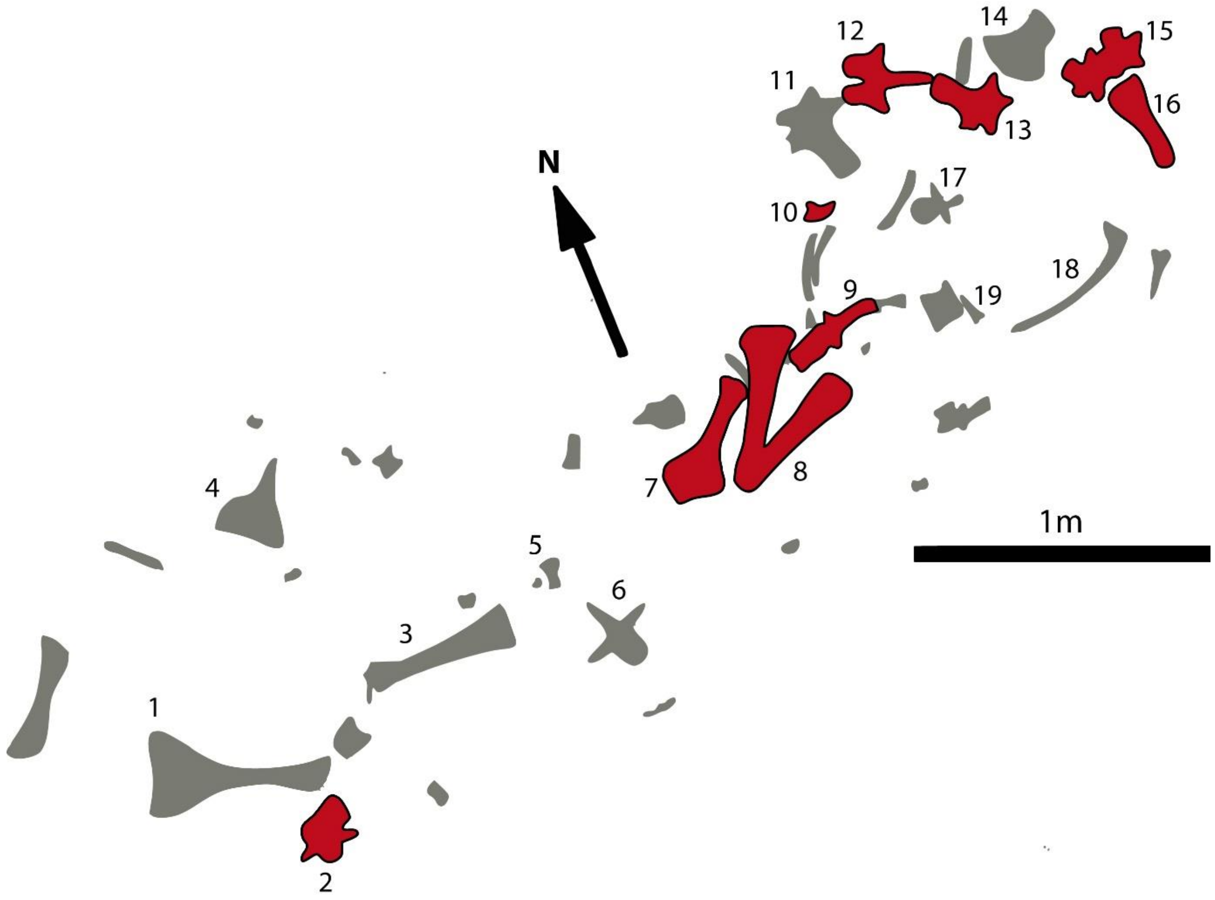

Numbers: 1, ischium (BP/1/6202); 2, anterior-to-middle cervical vertebra (BP/1/6199); 3, fibula (shattered upon extraction); 4, proximal ischium (BP/1/6184); 5, metacarpal IV (BP/1/6191); 6, mid-anterior caudal vertebra (BP/1/6201); 7, ischium (BP/1/7366); 8, paired tibiae (BP/1/6200 and BP/1/6980); 9, anterior caudal vertebra (BP/1/6646); 10, pedal ungual I (BP/1/6186); 11, posterior dorsal neural arch (BP/1/6183a); 12, anterior middle dorsal neural arch (BP/1/6183); 13, middle dorsal neural arch (BP/1/6770); 14, badly weathered ischial peduncle of ilium; 15, anterior dorsal neural arches (BP/1/6882 and BP/1/6984); 16, ulna (BP/1/6210); 17, mid-to-distal caudal vertebra (BP/1/7741); 18, dorsal rib (BP/1/6768); 19, chevron (BP/1/6205). Clavicle was recovered from plastered block in the vicinity of 9-12. Teeth were found close to the tibiae-ischium cluster. Red represents bones figured in Figure 3 of main article. Unnumbered elements were either indeterminate or unable to be located at the time of study. Quarry map by AMY, drawn in Inkscape (vers. 0.91).

3) Figure S2: Additional images of holotype (BP/1/6882). A, posterior view; B, dorsal view. Photographs by BWM.

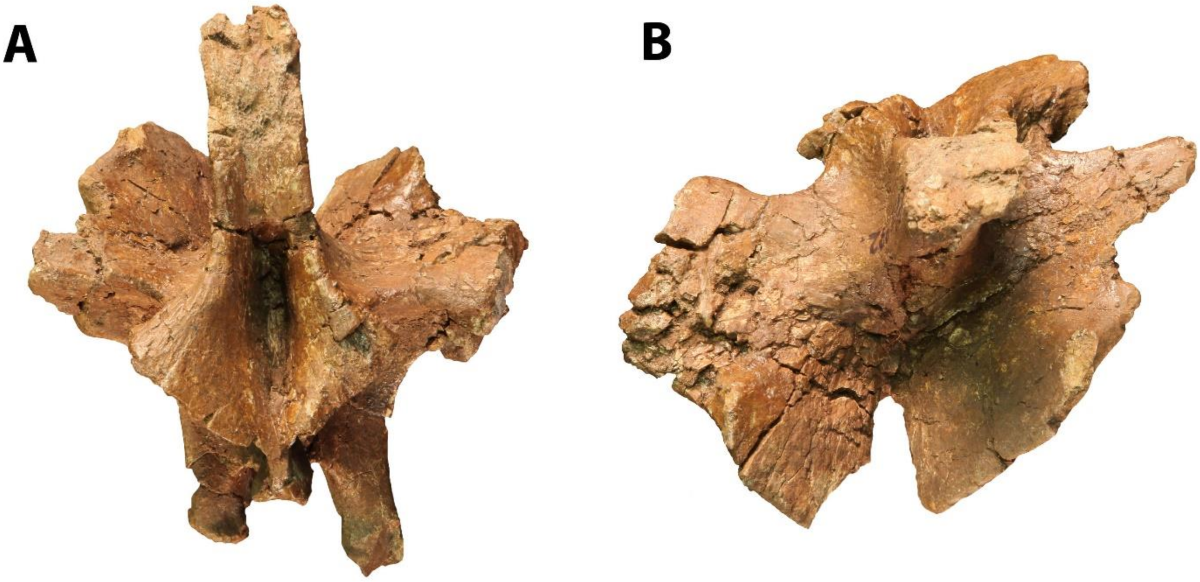

4) Figure S3: Spion Kop quarry. Tibiae and ischium *in situ.* Photograph by MFB.

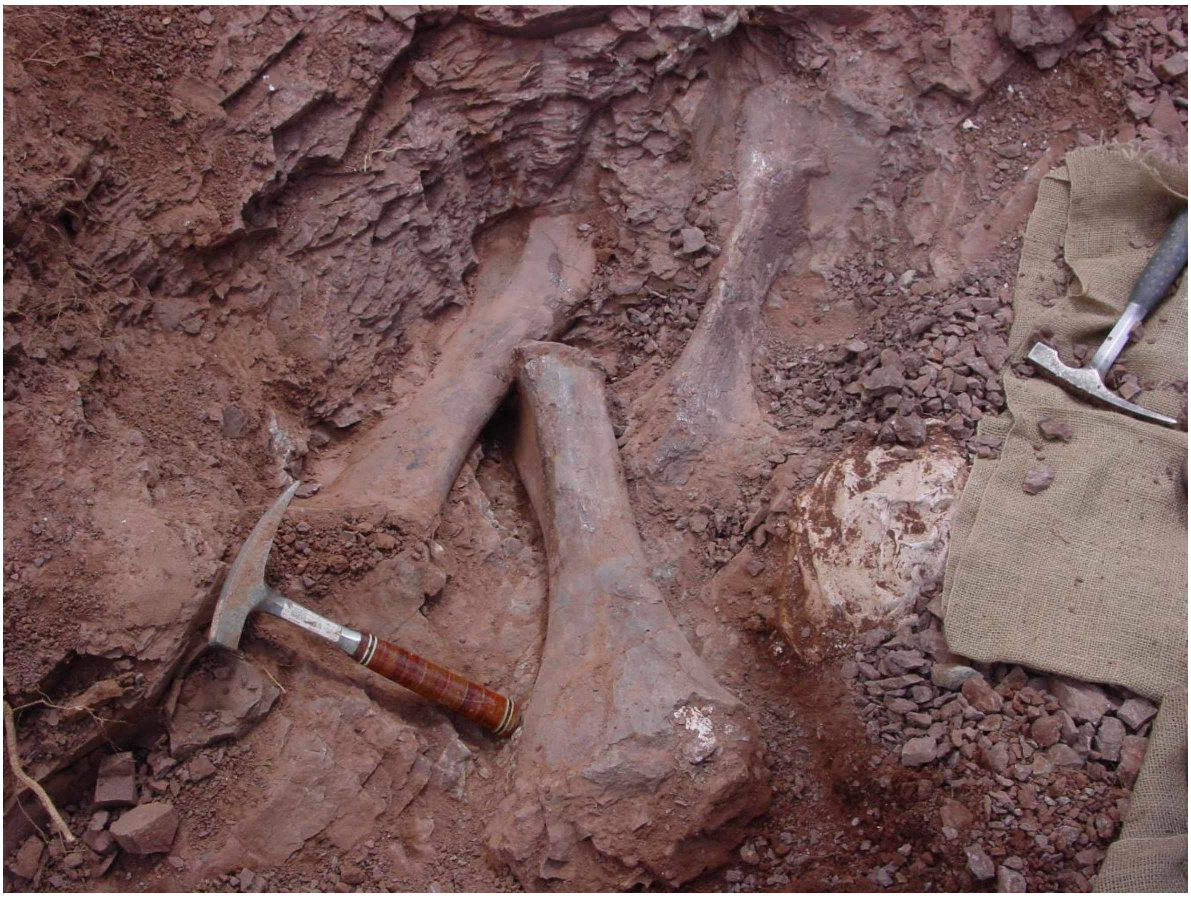

5) Supplementary Table S1: list of measurements

Anterior-to-middle cervical vertebra (BP/1/6199)	
Centrum: dorsoventral height of posterior face	70mm
Centrum: transverse width of posterior face	70mm
Maximum length of centrum (incomplete)	170mm
Maximum height of neural arch (including spine)	115mm
Anteroposterior length of dorsal margin of neural spine	50mm
Dorsoventral height of neural spine	55mm
Anterior-most dorsal arch (BP/1/6882)	
Anteroposterior length of neural spine base	35mm
Total dorsoventral height of neural arch (incomplete)	150mm
Anterior dorsal (BP/1/6984)	
Total dorsoventral height of neural arch (incomplete)	230mm
Total dorsoventral height of neural spine (incomplete)	120 mm
Anterior middle dorsal neural arch (BP/1/6183)	
Total dorsoventral height	220mm
Dorsoventral height of neural spine	125mm
Anteroposterior length of base of neural spine	75mm
Middle dorsal neural arch (BP/1/6770)	
Total dorsoventral height	225mm
Dorsoventral height of neural spine	140mm
Anteroposterior length of base of neural spine	80mm
Anterior caudal (BP/1/6646)	
Total dorsoventral height	395mm
Centrum: dorsoventral height of posterior face	160mm
Centrum: width of posterior face	145mm
Centrum: anteroposterior length of ventral surface	85mm
Anteroposterior length of base of neural spine	55mm
Dorosoventral height of neural spine	165mm
Clavicle (BP/1/6752)	
Maximum length	193mm
Humerus (BP/1/6193)	
Total proximodistal length (incomplete)	410mm
Ulna (BP/1/6210)	
Total proximodistal length (incomplete)	390mm
Metacarpal IV (BP/1/6191)	
Total proximodistal length	82mm
Transverse width of proximal surface	55mm
Maximum dorsoventral depth of proximal surface	33mm
Minimum transverse width of shaft	26mm
Transverse width of distal end	38mm
Left tibia (BP/1/6200)	
Total proximodistal length	520mm
Anteroposterior length of proximal end	240mm

Transverse width of proximal end	110mm
Maximum transverse width midshaft	99mm
Maximum anteroposterior depth midshaft	61mm
Right ischium (BP/1/7366)	
Total length (incomplete)	620mm
Anteroposterior length of proximal end (incomplete)	270mm
Pedal ungual I (BP/1/6186)	
Total proximodistal length	120mm
Transverse width proximal surface	38mm
Dorsoventral height proximal surface	65mm

6) Modifications to matrix of McPhee et al. (in press).

This analysis was based on a modified version of the character-by-taxon matrix employed in McPhee et al.[1] which was itself drawn from a successively modified version of the original data matrix of Yates[2] (see[3-8]).

In addition to *Pulanesaura*, the near-eusauropod *Spinophorosaurus* was added to the analysis and coded with reference to both the published literature[9,10] as well as its scorings within the original cladistic analysis of its relationships[10,11]. However, a large amount of anatomical information, clearly present within the well-preserved topotypic assemblage, remains unknown due to the preliminary nature of the single study thus far produced of its post-cranial morphology[10].

In addition to these taxa, three novel characters were introduced to the matrix. These are follows:

Character 187: Prezygadiapophyseal laminae on anterior caudal vertebrae: absent (0); present (1). As well as being present in *Pulanesaura*, the derived state of this character is advanced as a possible sauropodan synapomorphy.

Character 196: ‘Weaponized’ dermal spikes on tail: absent (0); present (1). This character is intended to homologise a morphology observed in both *Shunosaurus* and *Spinophorosaurus*.

Character 208: Shape of the humeral head: weakly developed, rounded in anterior-posterior view but minimally expanded perpendicular to the latter axis (0); flat in anterior-posterior view with only a slightly expanded lateral component (1); domed, being convex/hemispherical in anterior-posterior view with a strong lateral incursion onto the humeral shaft (2) (unordered). It was felt that the subtle variation present within the proximal surface of the primitive dinosaurian humerus was under-represented within the current data matrix. This character, in addition to building upon the observations of Remes[12], is intended to go some way towards correcting that

References

1 McPhee, B. W., Choiniere, J. N., Yates, A. M. & Viglietti, P. A. A Second Species of *Eucnemesaurus* Van Hoepen, 1920 (Dinosauria, Sauropodomorpha): new information on the diversity and evolution of the sauropodomorph fauna of South Africa's lower Elliot Formation (latest Triassic). *J Vertebr Paleontol* (In Press).

2 Yates, A. M. The first complete skull of the Triassic disnosaur *Melanorosaurus* Haughton (Sauropodomorpha: Anchisauria). *Special Papers in Paleontology* **77**, 9-55 (2007).

3 Apaldetti, C., Martinez, R. N., Alcober, O. A. & Pol, D. A New Basal Sauropodomorph (Dinosauria: Saurischia) from Quebrada del Barro Formation (Marayes-El Carrizal Basin), Northwestern Argentina. *PLoS ONE* **6**, e26964, doi:10.1371/journal.pone.0026964 (2011).

4 MartÍnez, R. N., Apaldetti, C. & Abelin, D. Basal sauropodomorphs from the Ischigualasto Formation. *J Vertebr Paleontol* **32**, 51-69 (2012).

5 McPhee, B. W., Yates, A. M., Choiniere, J. N. & Abdala, F. The complete anatomy and phylogenetic relationships of *Antetonitrus ingenipes* (Sauropodiformes, Dinosauria): implications for the origins of Sauropoda. *Zool J Linn Soc* **171**, 151-205, doi:10.1111/zoj.12127 (2014).

6 Otero, A. & Pol, D. Postcranial anatomy and phylogenetic relationships of *Mussaurus patagonicus* (Dinosauria, Sauropodomorpha). *J Vertebr Paleontol* **33**, 1138-1168 (2013).

7 Smith, N. D. & Pol, D. Anatomy of a basal sauropodomorph from the Early Jurassic Hanson Formation of Antarctica. *Acta Palaeontol Pol* **52**, 657-674 (2007).

8 Yates, A. M., Bonnan, M. F., Neveling, J., Chinsamy, A. & Blackbeard, M. G. A new transitional sauropodomorph dinosaur from the Early Jurassic of South Africa and the evolution of sauropod feeding and quadrupedalism. *Proc R Soc Biol Sci Ser B* **277**, 787-794, doi:10.1098/rspb.2009.1440 (2010).

9 Knoll, F., Witmer, L. M., Ortega, F., Ridgely, R. C. & Schwarz-Wings, D. The braincase of the basal sauropod dinosaur *Spinophorosaurus* and 3D reconstructions of the cranial endocast and inner ear. *PLoS ONE* **7**, e30060 (2012).

10 Remes, K. *et al.* A New Basal Sauropod Dinosaur from the Middle Jurassic of Niger and the Early Evolution of Sauropoda. *PLoS ONE* **4**, e6924, doi:10.1371/journal.pone.0006924 (2009).

11 Wilson, J. A. Sauropod dinosaur phylogeny: critique and cladistic analysis. *Zool J Linn Soc* **136**, 215-275, doi:10.1046/j.1096-3642.2002.00029.x (2002).

12 Remes, K. *Evolution of the Pectoral Girdle and Forelimb in Sauropodomorpha (Dinosauria, Saurischia): osteology, myology and function* PhD thesis, Ludwig-Maximilians-Universitat Munchen, (2008).

7) Explanation of ambiguous characters supporting a derived position of Pulanesaura:

117: More inclusive: present also in *Gongxianosaurus*.

119: Lack of information: unknown in *Vulcanodon* and *Isanosaurus* and possibly reversed in *Tazoudasurus*.

133: Inclusiveness and lack of information: Also present in *Lessemsaurus* (although only posterior cervical vertebrae are known for that taxon), while unknown for *Antetonitrus*, *Gongxianosaurus*, *Leonersaurus*, *Vulcanodon* and *Isanosaurus*.

186: Inclusiveness and lack of information: Reported for *Melanorosaurus*, unknown for *Isanosaurus* and *Tazoudasaurus*.

187: Lack of information: unknown for *Vulcanodon* and *Isanosaurus*.

213: Lack of information: unknown for *Gongxianosaurus*.

310: Inclusiveness and lack of information: Present if less pronounced in *Antetonitrus*, unknown for *Isanosaurus*.

315: Inclusiveness and lack of information: Shared with *Antetonitrus* and *Tazoudasaurus*, unknown for all other closely related taxa.

8) Character list used in analysis:

1. Skull to femur ratio: greater than 0.6 (0); less than 0.6 (1).

2. Lateral plates appressed to the labial side of the premaxillary, maxillary and dentary teeth: absent (0); present (1).

3. Relative height of the rostrum at the posterior margin of the naris: more than 0.6 the height of the skull at the middle of the orbit (0); less than 0.6 the height of the skull at the middle of the orbit (1).

4. Foramen on the lateral surface of the premaxillary body: absent (0); present (1).

5. Distal end of the dorsal premaxillary process: tapered (0); transversely expanded (1).

6. Profile of premaxilla: convex (0); with an inflection at the base of the dorsal process (1).

7. Size and position of the posterolateral process of premaxilla: large and lateral to the anterior process of the maxilla (0); small and medial to the anterior process of the maxilla (1).

8. Relationship between posterolateral process of the premaxilla and the anteroventral process of the nasal: broad sutured contact (0); point contact (1); separated by maxilla (2). Ordered.

9. Posteromedial process of the premaxilla: absent (0); present (1)

10. Shape of the anteromedial process of the maxilla: narrow, elongated and projecting anterior to lateral premaxilla-maxilla suture (0); short, broad and level with lateral premaxilla-maxilla suture (1).

11. Development of external narial fossa: absent to weak (0); well-developed with sharp posterior and anteroventral rims (1).

12. Development of narial fossa on the anterior ramus of the maxilla: weak and orientated laterally to dorsolaterally (0); well-developed and forming a horizontal shelf (1).

13. Size and position of subnarial foramen: absent (0); small (no larger than adjacent maxillary neurovascular foramina) and positioned outside of narial fossa (1); large and on the rim of, or inside, the narial fossa (2). Ordered.

14. Shape of subnarial foramen: rounded (0); slot-shaped (1).

15. Maxillary contribution to the margin of the narial fossa: absent (0); present (1).

16. Diameter of external naris: less than 0.5 of the orbital diameter (0); greater than 0.5 of the orbital diameter.

17. Shape of the external naris (in adults): rounded (0); subtriangular with an acute posteroventral corner (1).

18. Level of the anterior margin of the external naris: anterior to the midlength of the premaxillary body (0); posterior to the midlength of the premaxillary body (1).

19. Level of the posterior margin of external naris: anterior to, or level with the premaxilla-maxilla suture (0); posterior to the first maxillary alveolus (1); posterior to the midlength of the maxillary tooth row and the anterior margin of the antorbital fenestra (2). Ordered.

20. Dorsal profile of the snout: straight to gently convex (0); with a depression behind the naris (2).

21. Elongate median nasal depression: absent (0); present (1).

22. Width of anteroventral process of nasal at its base: less than the width of the anterodorsal process at its base (0); greater than the width of the anterodorsal process at its base (1).

23. Nasal relationship with dorsal margin of antorbital fossa: not contributing to the margin of the antorbital fossa (0); lateral margin overhangs the antorbital fossa and forms its dorsal margin (1); overhang extensive, obscuring the dorsal lachrymal-maxilla contact in lateral view (2). Ordered.

24. Pointed caudolateral process of the nasal overlapping the lachrymal: absent (0); present (1).

25. Anterior profile of the maxilla: slopes continuously towards the rostral tip (0); with a strong inflection at the base of the ascending ramus, creating a rostral ramus with parallel dorsal and ventral margins (1).

26. Length of rostral ramus of the maxilla: less than its dorsoventral depth (0); greater than its dorsoventral depth (1).

27. Shape of the main body of the maxilla: tapering posteriorly (0); dorsal and ventral margins parallel for most of their length (1).

28. Shape of the ascending ramus of the maxilla in lateral view: tapering dorsally (0); with an anteroposterior expansion at the dorsal end (1).

29. Rostrocaudal length of the antorbital fossa: greater than that of the orbit (0); less than that of the orbit (1).

30. Posteroventral extent of medial wall of antorbital fossa: reaching the anterior tip of the jugal (0); terminating anterior to the anterior tip of the jugal (1).

31. Development of the antorbital fossa on the ascending ramus of the maxilla: deeply impressed and delimited by a sharp, scarp-like rim (0); weakly impressed and delimited by a rounded rim or a change in slope (1).

32. Shape of the antorbital fossa: crescentic with a strongly concave posterior margin that is roughly parallel to the anterior margin of the antorbital fossa (0); subtriangular with a straight to gently concave posterior margin (1); antorbital fossa absent (2).

33. Size of the neurovascular foramen at the posterior end of the lateral maxillary row: not larger than the others (0); distinctly larger than the others in the row (1).

34. Direction that the neurovascular foramen at the posterior end of the lateral maxillary row opens: posteriorly (0); anteriorly, ventrally, or laterally (1).

35. Arrangement of lateral maxillary neurovascular foramina: linear (0); irregular (1).

36. Longitudinal ridge on the posterior lateral surface of the maxilla: absent (0); present (1).

37. Dorsal exposure of the lachrymal: present (0); absent (1).

38. Shape of the lachrymal: dorsoventrally short and blockshaped (0); dorsoventrally elongate and shaped like an inverted L (1).

39. Orientation of the lachrymal orbital margin: strongly sloping anterodorsally (0); erect and close to vertical (1).

40. Length of the anterior ramus of the lachrymal: greater than half the length of the ventral ramus (0); less than half the length of the ventral ramus (1); absent altogether (2). Ordered.

41. Web of bone spanning junction between anterior and ventral rami of lachrymal: absent and antorbital fossa laterally exposed (0); present, obscuring posterodorsal corner of antorbital fossa (1).

42. Extension of the antorbital fossa onto the ventral end of the lachrymal: present (0); absent (1).

43. Length of the posterior process of the prefrontal: short (0); elongated, so that total prefrontal length is equal to the anteroposterior diameter of the orbit (1).

44. Ventral process of prefrontal extending down the posteromedial side of the lachrymal: present (0); absent (1).

45. Maximum transverse width of the prefrontal: less than 0.25 of the skull width at that level (0); more than 0.25 of the skull width at that level (1).

46. Shape of the orbit: subcircular (0); ventrally constricted making the orbit subtriangular (1).

47. Slender anterior process of the frontal intruding between the prefrontal and the nasal: absent (0); present (1).

48. Jugal-lachrymal relationship: lachrymal overlapping lateral surface of jugal or abutting it dorsally (0); jugal overlapping lachrymal laterally (1).

49. Shape of the suborbital region of the jugal: an anteroposteriorly elongate bar (0); an anteroposteriorly shortened plate (1).

50. Jugal contribution to the antorbital fenestra: absent (0); present (1).

51. Dorsal process of the anterior jugal: present (0); absent (1).

52. Ratio of the minimum depth of the jugal below the orbit to the distance between the anterior end of the jugal and the anteroventral corner of the infratemporal fenestra: less than 0.2 (0); greater than 0.2 (1).

53. Transverse width of the ventral ramus of the postorbital: less than its anteroposterior width at midshaft (0); greater than its anteroposterior width at midshaft (1).

54. Shape of the dorsal margin of postorbital in lateral view: straight to gently curved (0); with a distinct embayment between the anterior and posterior dorsal processes (1).

55. Height of the postorbital rim of the orbit: flush with the posterior lateral process of the postorbital (0); raised so that it projects laterally to the posterior dorsal process (1).

56. Postfrontal bone: present (0); absent (1).

57. Position of the anterior margin of the infratemporal fenestra: behind the orbit (0); extends under the rear half of the orbit (1); extends as far forward as the midlength of the orbit (2). Ordered.

58. Frontal contribution to the supratemporal fenestra: present (0); absent (1).

59. Orientation of the long axis of the supratemporal fenestra: longitudinal (0); transverse (1).

60. Medial margin of supratemporal fossa: simple smooth curve (0); with a projection at the frontal/postorbital-parietal suture producing a scalloped margin (1).

61. Length of the quadratojugal ramus of the squamosal relative to the width at its base: less than four times its width (0); greater than four times its width (1).

62. Proportion of infratemporal fenestra bordered by squamosal: more than 0.5 of the depth of the infratemporal fenestra (0); less than 0.5 of the depth of the infratemporal fenestra (1).

63. Squamosal-quadratojugal contact: present (0); absent (1).

64. Angle of divergence between jugal and squamosal rami of quadratojugal: close to 90 degrees (0); close to parallel (1).

65. Length of jugal ramus of quadratojugal: no longer than the squamosal ramus (0); longer than the squamosal ramus (1).

66. Shape of the rostral end of the jugal ramus of the quadratojugal: tapered (0); dorsoventrally expanded (1).

67. Relationship of quadratojugal to jugal: jugal overlaps the lateral surface of the quadratojugal (0); quadratojugal overlaps the lateral surface of the jugal (1); quadratojugal sutures along the ventrolateral margin of the jugal (2).

68. Position of the quadrate foramen: on the quadrate-quadratojugal suture (0); deeply incised into, and partly encircled by, the quadrate (1); on the quadrate-squamosal suture, just below the quadrate head (2).

69. Shape of posterolateral margin of quadrate: sloping anterolaterally from posteromedial ridge (0); everted posteriorly creating a posteriorly facing fossa (1); posterior fossa deeply excavated, invading quadrate body (2). Ordered.

70. Exposure of the lateral surface of the quadrate head: absent, covered by lateral sheet of the squamosal (0); present (1).

71. Proportion of the length of the quadrate that is occupied by the pterygoid wing: at least 70 per cent (0); greater than 70 per cent (1).

72. Depth of the occipital wing of the parietal: less than 1.5 times the depth of the foramen magnum (0); more than 1.5 times the depth of the foramen magnum (1).

73. Position of foramina for mid-cerebral vein on occiput: between supraoccipital and parietal (0); on the supraoccipital (1).

74. Postparietal fenestra between supraoccipital and parietals: absent (0); present (1).

75. Shape of the supraoccipital: diamond-shaped, at least as high as wide (0); semilunate and wider than high (1).

76. Orientation of the supraoccipital plate: erect to gently sloping (0); strongly sloping forward so that the dorsal tip lies level with the basipterygoid processes (1).

77. Orientation of the paroccipital processes in occipital view: slightly dorsolaterally directed to horizontal (0); ventrolaterally directed (1).

78. Orientation of the paroccipital processes in dorsal view: posterolateral forming a V-shaped occiput (0); lateral forming a flat occiput (1)

79. Size of the post-temporal fenestra: large fenestra (0); a small hole that is much less than half the depth of the paroccipital process (1).

80. Exit of the mid-cerebral vein: through trigeminal foramen (0); through a separate foramen anterodorsal to trigeminal foramen (1).

81. Shape of the floor of the braincase in lateral view: relatively straight with the basal tuberae, basipterygoid processes and parasphenoid rostrum roughly aligned (0); bent with the basipterygoid processes and the parasphenoid rostrum below the level of the basioccipital condyle and the basal tuberae (1); bent with the basal tuberae lowered below the level of the basioccipital and the parasphenoid rostrum raised above it (2).

82. Shape of basal tuberae: knob-like, with basisphenoidal component rostral to basioccipital component (0); forming a transverse ridge with the basisphenoidal component lateral to the basioccipital component (1).

83. Length of the basipterygoid processes (from the top of the parasphenoid to the tip of the process): less than the height of the braincase (from the top of the parasphenoid to the top of the supraoccipital) (0); greater than the height of the braincase (from the top of the parasphenoid to the top of the supraoccipital) (1).

84. Ridge formed along the junction of the parabasisphenoid and the basioccipital, between the basal tuberae: present with a smooth anterior face (0); present with a median fossa on the anterior face (1); absent with the basal tuberae being separated by a deep posteriorly opening U-shaped fossa (2).

85. Deep septum spanning the interbasipterygoid space: absent (0); present (1).

86. Dorsoventral depth of the parasphenoid rostrum: much less than the transverse width (0); about equal to the transverse width (1).

87. Shape of jugal process of ectopterygoid: gently curved (0); strongly recurved and hook-like (1).

88. Pneumatic fossa on the ventral surface of the ectopterygoid: present (0); absent (1).

89. Relationship of the ectopterygoid to the pterygoid: ectopterygoid overlapping the ventral surface of the pterygoid (0); ectopterygoid overlapping the dorsal surface of the pterygoid (1).

90. Position of the maxillary articular surface of the palatine: along the lateral margin of the bone (0); at the end of a narrow anterolateral process due to the absence of the posterolateral process (1).

91. Centrally located tubercle on the ventral surface of palatine: absent (0); present (1).

92. Medial process of the pterygoid forming a hook around the basipterygoid process: absent (0); flat and blunt-ended (1); bent upward and pointed (2). Ordered.

93. Length of the vomers: less than 0.25 of the total skull length (0); more than 0.25 of the total skull length (1).

94. Position of jaw joint: no lower than the level of the dorsal margin of the dentary (0); depressed, well below this level (1).

95. Shape of upper jaws in ventral view: narrow with an acute rostral apex (0); broad and U-shaped (1).

96. Length of the external mandibular fenestra: more than 0.1 of the length of the mandible (0); less than 0.1 of the length of the mandible (1).

97. Caudal end of dentary tooth row medially inset with a thick lateral ridge on the dentary forming a buccal emargination : absent (0); present (1).

98. Height : length ratio of the dentary: less than 0.2; greater than 0.2 (1).

99. Orientation of the symphyseal end of the dentary: in line with the long axis of the dentary (0); strongly curved ventrally (1).

100. Position of first dentary tooth: adjacent to symphysis (0); inset one tooth's width from the symphysis (1).

101. Dorsoventral expansion at the symphyseal end of the dentary: absent (0); present (1).

102. Splenial foramen: absent (0); present and enclosed (1); present and open anteriorly (2). Ordered.

103. Splenial-angular joint: flattened sutured contact (0); synovial joint surface between tongue-like process of angular fitting in groove of the splenial (1).

104. A stout, triangular, medial process of the articular, behind the glenoid : present (0); absent (1).

105. Length of the retroarticular process: less than the depth of the mandible below the glenoid (0); greater than the depth of the mandible below the glenoid (2).

106. Strong medial embayment behind glenoid of the articular in dorsal view: absent (0); present (1).

107. Number of premaxillary teeth: four (0); more than four (1).

108. Number of dentary teeth (in adults): less than 18 (0); 18 or more (1).

109. Arrangement of teeth within the jaws: linearly placed, crowns not overlapping (0); imbricated with distal side of tooth overlapping mesial side of the succeeding tooth (1).

110. Orientation of the maxillary tooth crowns: erect (0); procumbent (1).

111. Orientation of the dentary tooth crowns: erect (0); procumbent (1).

112. Teeth with basally constricted crowns: absent (0); present (1).

113. Tooth-tooth occlusal wear facets : absent (0); present (1).

114. Mesial and distal serrations of the teeth: fine and set at right angles to the margin of the tooth (0); coarse and angled upwards at an angle of 45 degrees to the margin of the tooth (1).

115. Distribution of serrations on the maxillary and dentary teeth: present on both the mesial and distal carinae (0); absent on the posterior carinae (1); absent on both carinae (2).

116. Long axis of the tooth crowns distally recurved: present (0); absent (1).

117. Texture of the enamel surface: entirely smooth (0); finely wrinkled in some patches (1); extensively and coarsely wrinkled (2). Ordered.

118. Lingual concavities of the teeth: absent (0); present (1).

119. Longitudinal labial grooves on the teeth: absent (0); present (1).

120. Distribution of the serrations along the mesial and distal carinae of the tooth: extend along most of the length of the crown (0); restricted to the upper half of the crown (1).

121. Number of cervical vertebrae: eight or fewer (0); 9-10 (1); 12-13 (2); more than 13 (3). Ordered.

122. Shallow, dorsally facing fossa on the atlantal neurapophysis bordered by a dorsally everted lateral margin: absent (0); present (1).

123. Width of axial intercentrum: less than width of axial centrum (0); greater than width of axial centrum (1).

124. Position of axial prezygapophyses: on the anterolateral surface of the neural arch (0); mounted on anteriorly projecting pedicels (1).

125. Posterior margin of the axial postzygapophyses: overhang the axial centrum (0); flush with the caudal face of the axial centrum (1).

126. Length of the axial centrum: less than three times the height of the centrum (0); at least three times the height of the centrum (1).

127. Length of the anterior cervical centra (cervicals 3-5): no more than the length of the axial centrum (0); greater than the length of the axial centrum (1).

128. Length of middle to posterior cervical centra (cervical 6-8): no more than the length of the axial centrum (0); greater than the length of the axial centrum (1).

129. Dorsal excavation of the cervical parapophyses: absent (0); present (1).

130. Lateral compression of the anterior cervical vertebrae: centra are no higher than they are wide (0); are approximately 1.25 times higher than wide (1).

131. Relative elongation of the anterior cervical centra (cervical 3-5): lengths of the centra are less than 2.5 times the height of their anterior faces (0); lengths are 2.5-4 times the height of their anterior faces (1); the length of at least cervical 4 or 5 exceeds 4 times the anterior centrum height (2). Ordered.

132. Ventral keels on cranial cervical centra: present (0); absent (1).

133. Height of the mid cervical neural arches: no more than the height of the posterior centrum face (0); greater than the height of the posterior centrum face (1).

134. Cervical epipophyses on the dorsal surface of the postzygapophyses: absent (0); present on at least some cervical vertebrae (1).

135. Posterior ends of the anterior, postaxial epipophyses: with a free pointed tip (0); joined to the postzygapophysis along their entire length (1).

136. Shape of the epipophyses: tall ridges (0); flattened, horizontal plates (1).

137. Epipophyses overhanging the rear margin of the postzygapophyses: absent (0); present in at least some postaxial cervical vertebrae (1).

138. Anterior spur-like projections on mid-cervical neural spines: absent (0); present (1).

139. Shape of mid-cervical neural spines: less than twice as long as high (0); at least twice as long as high (1).

140. Shape of cervical rib shafts: short and posteroventrally directed (0); longer than the length of their centra and extending parallel to cervical column (1).

141. Position of the base of the cervical rib shaft: level with, or higher than the ventral margin of the cervical centrum (0); located below the ventral margin due to a ventrally extended parapophysis (1).

142. Postzygodiapophyseal lamina in cervical neural arches 4-8: present (0); absent (1).

143. Laminae of the cervical neural arches 4-8: well-developed tall laminae (0); weakly developed low ridges (1).

144. Shape of anterior centrum face in cervical centra: concave (0); flat (1); convex (2). Ordered.

145. Ventral surface of the centra in the cervicodorsal transition: transversely rounded (0); with longitudinal keels (1).

146. Number of vertebrae between cervicodorsal transition and primordial sacral vertebrae: 15-16 (0); no more than 14 (1).

147. Lateral surfaces of the dorsal centra: with at most vague, shallow depressions (0); with deep fossae that approach the midline (1); with invasive, sharp-rimmed pleurocoels (2). Ordered.

148. Oblique ridge dividing pleural fossa of cervical vertebrae: absent (0); present (1).

149. Laterally expanded tables at the midlength of the dorsal surface of the neural spines: absent in all vertebrae (0); present on the pectoral vertebrae (1); present on the pectoral and cervical vertebrae (2). Ordered.

150. Dorsal centra: entirely amphicoelous to amphiplatyan (1); first two dorsals are opisthocoelous (1); cranial half of dorsal column is opisthocoelous (2). Ordered.

151. Shape of the posterior dorsal centra: relatively elongated for their size (0); strongly axially compressed for their size (1).

152. Laminae bounding triangular infradiapophyseal fossae (chonae) on dorsal neural arches: absent (0); present (1).

153. Location of parapophysis in first two dorsals: at the anterior end of the centrum (0); located at the mid-length of the centrum, within the middle chonos (1).

154. Parapophyses of the dorsal column completely shift from the centrum to the neural arch: anterior to the thirteenth presacral vertebra (0); posterior to the thirteenth presacral vertebra (1).

155. Orientation of the transverse processes of the dorsal vertebrae: most horizontally directed (0); all upwardly directed (1).

156. Contribution of the paradiapophyseal lamina to the margin of the anterior chonos in mid-dorsal vertebrae: present (0); prevented by high placement of parapophysis (1).

157. Hyposphenes in the dorsal vertebrae: absent (0); present but less than the height of the neural canal (1); present and equal to the height of the neural canal (2). Ordered.

158. Prezygodiapophyseal lamina and associated anterior triangular fossa (anterior infradiapophyseal fossa): present on all dorsals (0); absent in mid-dorsals (1).

159. Anterior centroparapophyseal lamina in dorsal vertebrae: absent (0); present (1).

160. Prezygoparapophyseal lamina in dorsal vertebrae: absent (0); present (1).

161. Accessory lamina dividing posterior chonos from postzygapophysis: absent (0); present (1).

162. Pneumatic excavation of the dorsal neural arches: absent (0); equivocal (e.g., no more than depressions within the infradiapophyseal chambers) (1); sharp-rimmed subfossae or foramina clearly invading bone surface (2). Ordered.

163. Separation of lateral surfaces of anterior dorsal neural arches under transverse processes: widely spaced (0); only separated by a thin midline septum (1).

164. Height of dorsal neural arches, from neurocentral suture to level of zygapophyseal facets: much less than height of centrum (0); subequal to or greater than height of centrum (1).

165. Form of anterior surface of neural arch: simple centroprezygopophyseal ridge (0); broad anteriorly facing surface bounded laterally by centroprezygopophyseal lamina (1).

166. Shape of posterior dorsal neural canal: subcircular (0); slit-shaped (1).

167. Height of middle dorsal neural spines: less than the length of the base (0); higher than the length of the base but less than 1.5 times the length of the base (1); greater than 1.5 times the length of the base (2). Ordered.

168. Shape of anterior dorsal neural spines: lateral margins parallel in anterior view (0); transversely expanding towards dorsal end (2).

169. Cross-sectional shape of dorsal neural spines: transversely compressed (0); broad and triangular (1); square-shaped in posterior vertebrae (2). Ordered.

170. Spinodiapophyseal lamina on dorsal vertebrae: absent (0); present and separated from spinopostzygapophyseal lamina (1); present and joining spinopostzygapophyseal lamina to create a composite posterolateral spinal lamina (2). Ordered.

171. Well-developed, sheet-like suprapostzygapophyseal laminae: absent (0); present on at least the caudal dorsal vertebrae (2).

172. Shape of the spinopostzygapophyseal lamina in middle and posterior dorsal vertebrae: singular (0); bifurcated at its distal end

173. Shape of posterior margin of middle dorsal neural spines in lateral view: approximately straight (0); concave with a projecting posterodorsal corner (1).

174. Transversely expanded plate-like summits of posterior dorsal neural spines: absent (0); present (1).

175. Last presacral rib: free (0); fused to vertebra (1).

176. Sacral rib much narrower than the transverse process of the first primordial sacral vertebra (and dorsosacral if present) in dorsal view: absent (0); present (1).

177. Number of dorsosacral vertebrae: none (0); one (1); two (2). Ordered.

178. Caudosacral vertebra: absent (0); present (1).

179. Shape of the iliac articular facets of the first primordial sacral rib: singular (0); divided into dorsal and ventral facets separated by a non-articulating gap (1).

180. Deep, medially-directed pit excavating the surface of the non-articulating gap of the first primordial sacral rib: absent (0); present (1).

181. Depth of the iliac articular surface of the primordial sacrals: less than 0.75 of the depth of the ilium (0); greater than 0.75 of the depth of the ilium (1).

182. Sacral ribs contributing to the rim of the acetabulum: absent (0); present (1).

183. Posterior and anterior expansion of the transverse processes of the first and second primordial sacral vertebrae, respectively, partly roofing the intercostal space: absent (0); present (1).

184. Length of first caudal centrum: greater than its height (0); less than its height (1).

185. Position of postzygapophyses in proximal caudal vertebrae: protruding with an interpostzygapophyseal notch visible in dorsal view (0); placed on either side of the caudal end of the base of the neural spine without any interpostzygapophyseal notch (1).

186. A hyposphenal ridge on caudal vertebrae: absent (0); present (1).

187. Prezygadiapophyseal laminae on anterior caudals: absent (0); present (1).

188. Depth of the bases of the proximal caudal transverse processes: shallow, restricted to the neural arches (0); deep, extending from the centrum to the neural arch (1).

189. Position of last caudal vertebra with a protruding transverse process: distal to caudal 16 (0); proximal to caudal 16 (1).

190. Orientation of posterior margin of proximal caudal neural spines: sloping posterodorsally (0); vertical (1).

191. Longitudinal ventral sulcus on proximal and middle caudal vertebrae: present (0); absent (1).

192. Length of midcaudal centra: greater than twice the height of their anterior faces (0); less than twice the height of their anterior faces (1).

193. Cross-sectional shape of the distal caudal centra: oval with rounded lateral and ventral sides (0); square-shaped with flattened lateral and ventral sides (1).

194. Length of distal caudal prezygapophyses: short, not overlapping the preceding centrum by more than a quarter (0); long and overlapping the preceding the centrum by more than a quarter (1).

195. Shape of the terminal caudal vertebrae: unfused, size decreasing toward tip (0); expanded and fused to form a club-shaped tail (1).

196. 'Weaponized' dermal spikes on tail: absent (0); present (1).

197. Length of the longest chevron: less than twice the length of the preceding centrum (0); greater than twice the length of the preceding centrum (1).

198. Anteroventral process on distal chevrons: absent (0); present (1).

199. Mid-caudal chevrons with a ventral slit: absent (0); present (1).

200. Longitudinal ridge on the dorsal surface of the sterna plate: absent (0); present (1).

201. Craniocaudal length of the acromion process of the scapula: less than 1.5 times the minimum width of the scapula blade (0); greater than 1.5 times the minimum width of the scapula blade (1).

202. Minimum width of the scapula: greater than 20 per cent of its length (0); less than 20 per cent of its length (1).

203. Caudal margin of the acromion process of the scapula: rises from the blade at angle that is less than 65 degrees from the long axis of the scapula, at its steepest point (0); rises from the blade at angle that is greater than 65 degrees from the long axis of the scapula, at its steepest point (1).

204. Width of dorsal expansion of the scapula: less than the width of the ventral end of the scapula (0); equal to the width of the ventral end of the scapula (1).

205. Flat caudoventrally facing surface on the coracoids between glenoid and coracoid tubercle: absent (0); present (1).

206. Coracoid tubercle: present (0); absent (1).

207. Length of the humerus: less than 55 per cent of the length of the femur (0); 55-65 per cent of the length of the femur (1); 65-70 per cent of the length of the femur (2); more than 70 per cent of the length of the femur (3). Ordered.

208. Shape of the humeral head: weakly developed, rounded in anterior-posterior view but minimally expanded perpendicular to the latter axis (0); flat in anterior-posterior view with only a slightly expanded lateral component (1); domed, being convex/hemispherical in anterior-posterior view with a strong lateral incursion onto the humeral shaft (2) (Unordered).

209. Shape of the deltopectoral crest: subtriangular (0); subrectangular (1).

210. Length of the deltopectoral crest of the humerus: less than 30 per cent of the length of the humerus (0); 30-50 per cent of the length of the humerus (1); greater than 50 per cent of the length of the humerus (2). Ordered.

211. Shape of the anterolateral margin of the deltopectoral crest of the humerus: straight (0); strongly sinuous (1).

212. Rugose pit centrally located on the lateral surface of the deltopectoral crest: absent (0); present (1).

213. Well-defined fossa on the distal flexor surface of the humerus: present (0); absent (1).

214. Transverse width of the distal humerus: less than 33 per cent of the length of the humerus (0); greater than 33 per cent of the length of the humerus (1).

215. Shape of the entepicondyle of the distal humerus: rounded process (0): with a flat distomedially facing surface bounded by a sharp proximal margin (1).

216. Length of the radius: greater than 80 per cent of the humerus (0); less than 80 per cent of the humerus (1).

217. Deep radial fossa, bounded by an anterolateral process, on proximal ulna: absent (0); present (1).

218. Olecranon process on proximal ulna: present (0); absent (1); greatly enlarged olecranon (2).

219. Maximum linear dimensions of the ulnare and radiale: exceed that of at least one of the first three distal carpals (0); less than any of the distal carpals (1).

220. Transverse width of the first distal carpal: less than 120 per cent of the transverse width of the second distal carpal (0); greater than 120 per cent of the transverse width of the second distal carpal (1).

221. Sulcus across the medial end of the first distal carpal:absent (0); present (1).

222. Lateral end of first distal carpal: abuts second distal carpal (0); overlaps second distal carpal (1).

223. Second distal carpal: completely covers the proximal end of the second metacarpal (0); does not completely cover the proximal end of the second metacarpal (1).

224. Ossification of the fifth distal carpal: present (0); absent (1).

225. Length of the manus: less than 38 per cent of the humerus + radius (0); 38-45 per cent of the humerus + radius (1); greater than 45 per cent of the humerus + radius (2). Ordered.

226. Shape of metacarpus: flattened to gently curved and spreading (0); a colonnade of subparallel metacarpals tightly curved into a U-shape (1).

227. Proximal width of first metacarpal: less than the proximal width of the second metacarpal (0); greater than the proximal width of the second metacarpal (1).

228. Minimum transverse shaft width of first metacarpal: less than twice the minimum transverse shaft width of second metacarpal (0); greater than twice the minimum transverse shaft width of second metacarpal (1).

229. Proximal end of first metacarpal: flush with other metacarpals (0); inset into the carpus (1).

230. Shape of the first metacarpal: proximal width less than 65 per cent of its length (0); proximal width 65-80 per cent of its length (1); proximal width 80-100 per cent of its length (2); greater than 100 per cent of its length (3). Ordered.

231. Strong asymmetry in the lateral and medial distal condyles of the first metacarpal: absent (0); present (1).

232. Deep distal extensor pits on the second and third metacarpals: absent (0); present (1).

233. Shape of the distal ends of second and third metacarpals: subrectangular in distal view (0); trapezoidal with flexor rims of distal collateral ligament pits flaring beyond extensor rims (1).

234. Shape of the fifth metacarpal: longer than wide at the proximal end with a flat proximal surface (0); almost as wide as it is long with a strongly convex proximal articulation surface (1). (Yates ch.

235. Length of the fifth metacarpal: less than 75 per cent of the length of the third metacarpal (0); greater than 75 per cent of the length of the third metacarpal (1).

236. Length of manual digit one: less than the length of manual digit two (0); greater than the length of manual digit two (1).

237. Ventrolateral twisting of the transverse axis of the distal end of the first phalanx of manual digit one relative to its proximal end: absent (0); present but much less than 60 degrees (1); 60 degrees (2). Ordered.

238. Length of the first phalanx of manual digit one: less than the length of the first metacarpal (0); greater than the length of the first metacarpal (1).

239. Shape of the proximal articular surface of the first phalanx of manual digit one: rounded (0); with an embayment on the medial side (1).

240. Shape of the first phalanx of manual digit one: elongate and subcylindrical (0); strongly proximodistally compressed and wedge-shaped (1).

241. Length of the penultimate phalanx of manual digit two: less than the length of the second metacarpal (0); greater than the length of the second metacarpal (1).

242. Length of the penultimate phalanx of manual digit three: less than the length of the third metacarpal (0); greater than the length of the third metacarpal (1).

243. Shape of non-terminal phalanges of manual digits two and three: longer than wide (0); as long as wide (1).

244. Shape of the unguals of manual digits two and three: straight (0); strongly curved with tips projecting well below flexor margin of proximal articular surface (1).

245. Length of the ungual of manual digit two: greater than the length of the ungual of manual digit one (0); 75-100 per cent of the ungual of manual digit one (1); less than 75 per cent of the ungual of manual digit one (2); the ungual of manual digit two is absent (3). Ordered.

246. Phalangeal formula of manual digits two and three: three and four, respectively (0); with at least one phalanx missing from each digit (1).

247. Phalangeal formula of manual digits four and five: greater than 2-0, respectively (0); less than 2-0, respectively (1).

248. Strongly convex dorsal margin of the ilium: absent (0); present (1).

249. Cranial extent of preacetabular process of ilium: does not project further anterior than the anterior margin of the pubic peduncle (0); projects anterior to the cranial margin of the pubic peduncle (1).

250. Shape of the preacetabular process: blunt and rectangular (0); with a pointed, projecting anteroventral corner and a rounded dorsum (1).

251. Depth of the preacetabular process of the ilium: much less than the depth of the ilium above the acetabulum (0); subequal to the depth of the ilium above the acetabulum (1).

252. Length of preacetabular process of the ilium: less than twice its depth (0); greater than twice its depth (1).

253. Buttress between preacetabular process and the supraacetabular crest of the ilium: present (0); absent (1).

254. Medial wall of acetabulum: fully closing acetabulum with a triangular ventral process between the pubic and ischial peduncles (0); partially open acetabulum with a straight ventral margin between the peduncles (1); partially open acetabulum with a concave ventral

margin between the peduncles (2); fully open acetabulum with medial ventral margin closely approximating lateral rim of acetabulum (3). Ordered.

255. Length of the pubic peduncle of the ilium: less than twice the anteroposterior width of its distal end (0); greater than twice the anteroposterior width of its distal end .

256. Caudally projecting 'heel' at the distal end of the ischial peduncle: absent (0); present (1).

257. Length of the ischial peduncle of the ilium: similar to pubic peduncle (0); much shorter than pubic peduncle (1); virtually absent so that the chord connecting the distal end of the pubic peduncle with the ischial articular surface contacts the postacetabular process (2). Ordered.

258. Length of the postacetabular process of the ilium: between 40 and 100 per cent of the distance between the pubic and ischial peduncles (0); less than 40 per cent of the distance between the pubic and ischial peduncles (1); more than 100 per cent of the distance between the pubic and ischial peduncles (2).

259. Well-developed brevis fossa with sharp margins on the ventral surface of the postacetabular process of the ilium: absent (0); present, ventrally facing (1); present, lateroventrally facing (2). 256; 3rd state (2) from Ezcurra 2

260. Anterior end of ventrolateral ridge bounding brevis fossa: not connected to supracetabular crest (0); joining supracetabular crest (1).

261. Shape of the caudal margin of the postacetabular process of the ilium: rounded to bluntly pointed (0); square ended (1); with a pointed ventral corner and a rounded caudodorsal margin (2).

262. Width of the conjoined pubes: less than 75 per cent of their length (0); greater than 75 per cent of their length (1).

263. Pubic tubercle on the lateral surface of the proximal pubis: present (0); absent (1).

264. Proximal anterior profile of pubis: anterior margin of pubic apron smoothly confluent with anterior margin of iliac pedicel (0); iliac pedicel set anterior to the pubic apron creating a prominent inflection in the proximal anterior profile of the pubis (1).

265. Minimum transverse width of the pubic apron: much more than 40 per cent of the width across the iliac peduncles of the ilium (0); less than 40 per cent of the width across the iliac peduncles of the ilium (1). (Yates, 2007; - ch.

266. Position of the obturator foramen of the pubis: at least partially occluded by the iliac pedicel in anterior view (0); completely visible in anterior view (1).

267. Lateral margins of the pubic apron in anterior view: straight (0); concave (1).

268. Orientation of distal third of the blades of the pubic apron: confluent with the proximal part of the pubic apron (0); twisted posterolaterally relative to proximal section so that the anterior surface turns to face laterally (1).

269. Orientation of the entire blades of the pubic apron: transverse (0); twisted posteromedially (1).

270. Craniocaudal expansion of the distal pubis: absent (0); less than 15 per cent of the length of the pubis (1); greater than 15 per cent of the length of the pubis (2). Ordered.

271. Notch separating posteroventral end of the ischial obturator plate from the ischial shaft: present (0); absent (1).

272. Elongate interischial fenestra: absent (0); present (1).

273. Longitudinal dorsolateral sulcus on proximal ischium: absent (0); present (1).

274. Shape of distal ischium: broad and plate-like, not distinct from obturator region (0); with a discrete rod-like distal shaft (1).

275. Length of ischium: less than that of the pubis (0); greater than that of the pubis (1).

276. Ischial component of acetabular rim: larger than the pubic component (0); equal to the pubic component (1)

277. Shape of the transverse section of the ischial shaft: ovoid to subrectangular (0); triangular (1).

278. Orientation of the long axes of the transverse section of the distal ischia: meet at an angle (0); are coplanar (1).

279. Depth of the transverse section of the ischial shaft: much less than the transverse width of the section (0); at least as great as the transverse width of the section (1).

280. Distal ischial expansion: absent (0); present (1).

281. Transverse width of the conjoined distal ischial expansions: greater than their sagittal depth (0); less than their sagittal depth (1).

282. Length of the hindlimb: greater than the length of the trunk (0); less than the length of the trunk (1).

283. Longitudinal axis of the femur in lateral view: strongly bent with an offset between the proximal and distal axes greater than 15 degrees (0); weakly bent with an offset of less than 10 degrees (1); straight (2). Ordered.

284. Shape of the cross-section of the mid-shaft of the femur: subcircular (0); strongly elliptical with the long axis orientated mediolaterally (1).

285. Angle between the long axis of the femoral head and the transverse axis of the distal femur: about 30 degrees (0); close to 0 degrees (1).

286. Shape of femoral head: roughly rectangular in profile with a sharp medial distal corner (0); roughly hemispherical with no sharp medial distal corner (1). This character only applies to taxa with a medially, or anteromedially protruding femoral head. It does not apply to

outgroup taxa (Euparkeria or Crurotarsi) with proximally directed femoral heads and is coded as unknown in these taxa.

287. Posterior proximal tubercle on femur: well-developed (0); indistinct to absent (1).

288. Shape of the lesser trochanter: small rounded tubercle (0); proximodistally orientated, elongate ridge (1); absent (2).

289. Position of proximal tip of lesser trochanter: level with the femoral head (0); distal to the femoral head (1).

290. Projection of the lesser trochanter: just a scar upon the femoral surface (0); a raised process (1).

291. Transverse ridge extending laterally from the lesser trochanter: absent (0); present (1).

292. Height of the lesser trochanter in cross section: less than its basal width (0); at least as high as its basal width (1).

293. Position of the lesser trochanter in anterior view: near the centre of the anterior face of the femoral shaft (0); close to the lateral margin of the femoral shaft (1).

294. Visibility of the lesser trochanter in posterior view: not visible (0); visible (1).

295. Height of the fourth trochanter: a low rugose ridge (0); a tall crest (1).

296. Position of the fourth trochanter along the length of the femur: in the proximal half (0); straddling the midpoint (1).

297. Symmetry of the profile of the fourth trochanter of the femur: subsymmetrical without a sharp distal corner (0); asymmetrical with a steeper distal slope than the proximal slope and a distinct distal corner (1).

298. Shape of the profile of the fourth trochanter of the femur: rounded (0); subrectangular (1).

299. Position of fourth trochanter along the mediolateral axis of the femur: centrally located (0); on the medial margin (1).

300. Extensor depression on anterior surface of the distal end of the femur: absent (0); present (1).

301. Size of the medial condyle of the distal femur: subequal to the fibular + lateral condyles (0); larger than the fibular + lateral condyles (1).

302. Well-developed tibiofibular crest on distal femur: absent (0); present (1).

303. Distal surface of tibiofibular crest: as deep anteroposteriorly as wide mediolaterally or deeper (0); wider mediolaterally than deep anteroposteriorly (1). (Smith and Pol 2007 ch.

304. Tibia:femur length ratio: greater than 1.0 (0); between 0.6 and 1.0 (1); less than 0.6 (2). Ordered.

305. Orientation of cnemial crest: projects anteriorly to anterolaterally (0); projecting laterally (1).

306. Paramarginal ridge on lateral surface of cnemial crest: absent (0); present (1).

307. Position of the tallest point of the cnemial crest: close to the proximal end of the crest (0); about half-way along the length of the crest, creating an anterodorsally sloping proximal margin of the crest (1).

308. Proximal end of tibia with a flange of bone that contacts the fibula: absent (0): present (1).

309. Position of the posterior end of the fibular condyle on the proximal articular surface tibia: anterior to the posterior margin of the proximal articular surface (0); level with the posterior margin of the proximal articularsurface (1).

310. Shape of the proximal articular surface of the tibia: transverse width subequal to anteroposterior length (0); transverse width between 0.6 and 0.9 times anteroposterior length (1); anteroposterior length twice the transverse width or higher (2). Ordered.

311. Transverse width of the distal tibia: subequal to its craniocaudal length (0); greater than its craniocaudal length (1).

312. Anteroposterior width of the lateral side of the distal articular surface of the tibia: as wide as the anteroposterior width of the medial side (0); narrower than the anteroposterior width of the medial side (1).

313. Relationship of the posterolateral process of the distal end of the tibia with the fibula: not flaring laterally and not making significant contact with the fibula (0); flaring laterally and backing the fibula (1).

314. Shape of the distal articular end of the tibia in distal view: ovoid (0); subrectangular (1).

315. Shape of the anteromedial corner of the distal articular surface of the tibia: forming a right angle (0); forming an acute angle (1).

316. Position of the lateral margin of descending caudoventral process of the distal end of the tibia: protrudes laterally at least as far as the anterolateral corner of the distal tibia (0); set well back from the anterolateralcorner of the distal tibia (1).

317. A triangular rugose area on the medial side of the fibula: absent (0); present (1).

318. Transverse width of the midshaft of the fibula: greater than 0.75 of the transverse width of the midshaft of the tibia (0); between 0.5 and 0.75 of the transverse width of the midshaft of the tibia (1); less than 0.5 of thetransverse width of the midshaft of the tibia (2). Ordered.

319. Position of fibula trochanter: on anterior surface of fibula (0); laterally facing (1); anteriorly facing but with strong lateral bulge (2).

320. Depth of the medial end of the astragalar body in cranial view: roughly equal to the lateral end (0); much shallower creating a wedge-shaped astragalar body (1).

321. Shape of the posteromedial margin of the astragalus in dorsal view: forming a moderately sharp corner of a subrectangular astragalus (0); evenly rounded without formation of a caudomedial corner (1).

322. Dorsally facing horizontal shelf forming part of the fibular facet of the astragalus: present (0); absent with a largely vertical fibular facet (1).

323. Pyramidal dorsal process on the posteromedial corner of the astragalus: absent (0); present (1).

324. Shape of the ascending process of the astragalus: anteroposteriorly deeper than transversely wide (0); transversely wider than anteroposteriorly deep (1).

325. Posterior extent of ascending process of the astragalus: positioned anteriorly upon the astragalus (0); close to the posterior margin of the astragalus (1).

326. Sharp medial margin around the depression posterior to the ascending process of the astragalus: absent (0); present (1).

327. Buttress dividing posterior fossa of astragalus and supporting ascending process: absent (0); present (1).

328. Vascular foramina set in a fossa at the base of the ascending process of the astragalus: present (0); absent (1).

329. Distal articular surface of astragalus: relatively flat or weakly convex (0); extremely convex and roller-shaped (1).

330. Transverse width of the calcaneum: greater than 30 per cent of the transverse width of the astragalus (0); less than 30 per cent of the transverse width of the astragalus (1).

331. Lateral surface of calcaneum: simple (0); with a fossa (1).

332. Medial peg of calcaneum fitting into astragalus: present, even if rudimentary (0); absent (1).

333. Calcaneal tuber: large and well developed (0); highly reduced to absent (1).

334. Shape of posteromedial heel of distal tarsal four (lateral distal tarsal): proximodistally deepest part of the bone (0); no deeper than the rest of the bone (1).

335. Shape of posteromedial process of distal tarsal four in proximal view: rounded (0); pointed (1).

336. Ossified distal tarsals: present (0); absent (1).

337. Proximal width of the first metatarsal: less than the proximal width of the second metatarsal (0); at least as great as the proximal width of the second metatarsal (1).

338. Size of first metatarsal: maximum proximal breadth less than 0.4 times its proximodistal length (0); maximum proximal breadth between 0.4 and 0.7 times its proximodistal length (1); maximum proximal breadth greater than 0.7times its proximodistal length (2). Ordered.

339. Orientation of proximal articular surface of metatarsal one: horizontal (0); sloping proximolaterally relative to the long axis of the bone (1).

340. Shaft of metatarsal I: closely appressed to metatarsal II throughout its length (0); only closely appressed proximally, with a space between metatarsals I and II distally (1).

341. Orientation of the transverse axis of the distal end of metatarsal one: horizontal (0); angled proximomedially (1).

342. Shape of the medial margin of the proximal surface of the second metatarsal: straight (0); concave (1).

343. Shape of the lateral margin of the proximal surface of the second metatarsal: straight (0); concave (1).

344. Projection of ventral flange on proximal surface of second metatarsal: neither corner appreciably more developed than the other (0); laterally flaring (1); medially flaring (2).

345. Well-developed facet on proximolateral corner of plantar ventrolateral flange of mt II for articulation with medial distal tarsal: absent (0); present (1).

346. Length of the third metatarsal: greater than 40 per cent of the length of the tibia (0); less than 40 per cent of the length of the tibia (1).

347. Proximal outline of metatarsal III: subtriangular with acute or rounded posterior border (0); subtrapezoidal, with posterior border broadly exposed in plantar view (1).

348. Minimum transverse shaft diameters of third and fourth metatarsals: greater than 60 per cent of the minimum transverse shaft diameter of the second metatarsal (0); less than 60 per cent of the minimum transverse shaft diameter ofthe second metatarsal (1).

349. Transverse width of the proximal end of the fourth metatarsal: less than twice the anteroposterior depth of the proximal end (0); at least twice the anteroposterior depth of the proximal end (1).

350. Angle formed by the anterior and anteromedial borders of metatarsal IV: obtuse (0); right angle, or acute (1).

351. Transverse width of the proximal end of the fifth metatarsal: less than 25 percent of the length of the fifth metatarsal (0); between 30 and 49 percent of the length of the fifth metatarsa (1); greater than 50 percent of the length of the fifth metatarsal(2). Ordered.

352. Transverse width of distal articular surface of metatarsal four in distal view: greater than the anteroposterior depth (0); less than the anteroposterior depth (1).

353. Pedal digit five: reduced, non-weight bearing (0); large (fifth metatarsal at least 70 per cent of fourth metatarsal), robust and weight bearing (1).

354. Length of non-terminal pedal phalanges: all longer than wide (0); proximalmost phalanges longer than wide while more distal phalanges are as wide as long (1); all non-terminal phalanges are as wide, if not wider, than long(2).Ordered.

355. Length of the first phalanx of pedal digit one: greater than the length of the ungual of pedal digit one (0); less than the length of the ungual of pedal digit one (1).

356. Length of the ungual of pedal digit one: less than at least some non-terminal phalanges (0); longer than all non-terminal phalanges but shorter than first metatarsal (1); longer than the first metatarsal (2). Ordered.

357. Shape of the ungual of pedal digit one: shallow, pointed, with convex sides and a broad ventral surface (0); deep, abruptly tapering, with flattened sides and a narrow ventral surface (1).

358. Shape of proximal articular surface of pedal unguals: proximally facing, visible on medial and lateral sides (0); proximomedially facing and visible only in medial view, causing medial deflection of pedal unguals in articulation (1).

359. Penultimate phalanges of pedal digits two and three: well-developed (0); reduced disc-shaped elements if they are ossified at all (1).

360. Shape of the unguals of pedal digits two and three: dorsoventrally deep with a proximal articulating surface that is at least as deep as it is wide (0); dorsoventrally flattened with a proximal articulating surface that iswider than deep (1).

361. Length of the ungual of pedal digit two: greater than the length of the ungual of pedal digit one (0); between 90 and 100 per cent of the length of the ungual of pedal digit one (1); less than 90 per cent of the length of the ungual of pedal digit one (2). Ordered.

362. Size of the ungual of pedal digit three: greater than 85 per cent of the ungual of pedal digit two in all linear dimensions (0); less than 85 per cent of the ungual of pedal digit two in all linear dimensions (1).

363. Number of phalanges in pedal digit four: four (0); fewer than four (1).

364. Phalanges of pedal digit five: present (0); absent (1).

365. Femoral length: less than 200 mm (0); between 200 and 399 mm (1); between 400 and 599 mm (2); between 600 and 799 mm (3); between 800 and 1000 mm (4); greater than 1000 mm (5). Ordered.

9) Character-by-taxon matrix

Euparkeria

00000000?0000?0000000?000?1001000000000000000000?0000000001001000000000000
000?0000000?00?000?000000000?1000000000000000000?00000000000???0000??000000000?00?
0?00??0000000000000000000000?00?0?????0????000?00200000000000??????0000000001?0?
0?0000???00?0010?0000?0110000000000001000000000?02?000??0100000??1?000?100000?0000
000??0?0?0010??0000?0????0?00?1?100000000?000

Crurotarsi

0000000000000?0[01]000000000?0001000000[01]00000000000000000000001000000000000
0000000?0000000000000000000000000000000000000000000000000000???0000??00000200
0?00?0?00??00002000000000000000000000000000000[01]00?0[01]0?002?00000000000000000000
0000000000000000[01]000000010?0000?0[01]0000000000000000000000?00?0000000000000-
1?0000[01]00000?0000000??0?0000000000?00000000?00?00100000000000[01]

Marasuchus

00???????0?0????????????0?00???????0???????????0???????????????????????????00???
00000???????????????????????00?00000000?1?100000000010???00?0??00?000000?00???00??00
0?00000000?000000000??0010100000000?000?012?1000?0000?????????????????????????????0
00000000000?0100010000000000000000100000001100010000000??000000100010001001000000?0?
0000000000?000??0?00?0?00?0??00???10

Aardonyx

1101?00210002?11?11?????1110?1011000??????1010??0?11010??10?1????????1?0?????
???????0??????1??0?0?1?0?????11?00101011001?1??????0010?110100101101?0000011?001100
01000020000010?1100??0110000?01110?0?00?????11??????????00??00??????13101???1?10??1
0?????????????????1110100010011??10110?101?1111000011110001????????1101110?0???01???
????????01201111?0?0010?0?2??00?0???03

Adeopapposaurus

100110021000211101111101101111101000010110100011011111111001000102001[01]0
110010110101010110100000101[01]10101011111110[01]100111001111002001000111011011100
2001110011000000000000000001001101?0010000000001000000000100101211000111001101111011
2101100111000002000010003110010001101100 10?11001011000000110100001021010101000001
1101100100111101000011101000011110000010200011000011001

Anchisaurus

10???00??0102?1??11?????111011?010?001010?10001100111101100?10??????01?00110
1?1??012110??????10?10?000?10000000101?1[12]?0111?0011???1001001111011?10000?010?00
1100?000??0000000??01000?0???001?00?10?0????11[01]0??2?1100101100??0??1101011100100
201000002000[01]10103100000?1111100010011?0010100001011010000 10[01]1110?0100000111
0111?1?01?010????10111?0?0011????0?01?1?1010000010011

Antetonitrus

??
???0?[01]0???????????00?000001??00[12]10002
010121001000???1?????10?00?01100?0??0?0011??3211000[01]0110??????0010?3100???1010???

????0010003???0002010010001????????????1101111001111111110?1100000210010101010???????
??????????121111120100?????[12]1200?0????[34]

Barapasaurus

??
??????????????????????????????1?111211???????????1?1????00??002??100101??002011021111
20121100?0[12]10001?1?1?1??1????0????101?00?21???????11??????????????????????????????11
11013101100011111001 1???11110010???????????????????1???1?1?????????1?1???????1???????
???20?????????????????2?1??????5

Blikanasaurus

??
???
???
??0000?1110111?1?0100101101101110 0120111
1001?1112102120000100?2

Cetiosaurus

1??11?????????????????????????
???2??0011110111110100100020?2001?1??1020??1[12]1
1102010100???[12]??????10111?00100?0??0111101032110010001 1???????1???????????????????
??1111013?0210?0110?1?011??1101?00???211112??0???010?111??210100210010111??????????
?????????????????????????????????????5

Chindesaurus

??
???0??????????????01?0??0?1??0?1??????000??
00?????000????1?0?????1000?0???0
???001??????0??????????????0000101110001011 1000??????11111100???01?110100???????????
??????????????????????????1

Chromogisaurus

??
???
????????10000??10?0??1??????????????????????????0??????????????????????????????0????1110
02202??????????????????????0??????1???10110????100000[12]010100010?????????????????????
???????????0??0???000???10

Coloradisaurus

?00?1002??102?111111?1??1110??011000?10??01?10?1011101110000100100?10100110
1101?110101?????1?1001011010011??000101010001?11?0011002001001 01?01100?00?001?1001
100000000000000101?????00?01000?011???0????0100101212100 11?????????????????1????????
???????0?0?131100???011?1100200110010111000001101000010211101110000011101100??0111
10?001??????000011111101010200011000021002

Efraasia

100?1001?010??1?111?112?1110?100???00100100000??0?10??01?0??1??????10?000?1?
????0110100??????10010010??11?0110010101000 01???1?1100110100101101101000 0001??001?
000000000000000001?00000100000?01100000?0?0100112012000111001101012010?1100000111
0100010000100131000101000010000001100101101000011110000101100000010000011101?001?0
11010?0??01?111000001??0?0?01?1?001[01]000010002

Eoraptor

0001?001?01010?00110001110100000100001101000000?10110010100?01100001001 1??11?1????????????????00000010???0?0?00?1000000001?????11000000000?1011??000??00?00???00?000??00000011??10????10???001?00??0000?0100??10?10??00100??????20?00011?00010?000001011010012000 2201??0??00?0???10?1011?10??0?011000010110001?00?1?0111010??1?0??0?????001????0?000?????0?0??0010000000??000

Eucnemesaurus

??0???01??001100010000??00?0????????????10000?01100???????????11??110???0???????????????01101?1010?1010110???000001110110???[23]

Glacialisaurus

??0????????????????0011????????????????0???1010[01]0????1100?0111111?1010???????????????

Gongxianosaurus

1?????0??????????12???00???1??2121????????????0????????????1??0??001??0??????0?0??00000????????????????0?0?1?0??000?011???2?110????1?1?????????????????????????????0?100????0??????????????????????????[12]111?2??????0???110??1??????1?0??1???0?1?1?????1?11??01?0??????1?0??2?1112000020005

Guaibasaurus

??0???01??011001000000??00000??0000000?00000?01?0??0[01]00??1??????110????1?0???????0????110??0?1?00??0?0?000?01[12]10022011001000000000010?10110000001001000010110 10??100?0?1111110 0100?01101?000101110000000000000001?011000000000011

Herrerasaurus

00000000?0101000000000000?0001000000000001000000100010101010001000011010000 00101?0000000?1000?00000000?1000000000000000000101000100000010000 0?000010000011??0010[01]100000020000001000000101100100110010010 0?1110??00110000000000000012000000100000110110100100000120000000100000 10???01?01010010000101110001011000001000011000100010010010100000001100000100000000100010000000000?02

Isanosaurus

??0??0???????????2??10??0?????????????????2?011000?????????????????????????101?0???211112??0???001?1?011?????????????????????????1????????????????0?????????????????????3

Jingshanosaurus

1001?002??10211111100?0?111011101100?102001??1?101110111000010 00????0110000

0101?211001000??1?10110010??11101100101211001????111??010??????1???10100??0011100?1
???0000000000000??10???01000000001000000?0010??121201011100??00??0011?3100100?010
0010200001001310000020110010O2??110010110?01001101000010111110??1010001110110?1201
1010?00???????0120?1112?1?01?2000120000010?4

Leonerasaurus

?0??
?????????????????0?0???????1?11010110011???001??010?1???????1100?00?0?10000110000000
??00000100?111?001?????????????????0100????1100000??????????????????????????????????11
0103100?????????0?????11??1?1????0???1
00111020????????????0???????

Lessemsaurus

???
??0?011??0?00???101?0000011100[12]1000000
0111000000??????????????????????????0001???21110010110???????0???310?????1?0??1????00
10013100000?011?10001???1??10110?111011?10???11111?0???00000[01]1?01?1???0110110011
??????????1?11?????1?2101??00??0???3

Leyesaurus

?001?00?1000211??11?????1010?11010000101101?00?1011111111?00100010[0
2]0011?????????01?10?110??1?00?10110??1010?1111010[01]0001?1???11100200100111011???
?0???????????????????????????????????00000?001????000??????????????????????????????????
???????????????????????????????????100???
????????????010??????????0?102000????0???0??

Lufengosaurus

100???02???0211?1?11?1111111111010010101101000010111010110001 0?1????0110??0
110??01010100?011010010????0111?11011010100011?000111002001101111?0110100020011100
1100000000000000001001101?0010000000[01]1000000000110111212[01]10111001101111011131O
010120100010200001001311000020110110021011001011000010110100001021110111010001111
01100100111110?00001?11100[01]011110101010200011000O[12][01]004

Mamenchisaurus

11000113?111201101200000000101112??0011120101010111111101211000001112210100
00111?201?00?????2?11[01]000010010001111111111211113?0001110121010000110002?11101010
10020110?011020121000001100111111111011001?011111100032210010011011001101100010000
11?0?10?1?31?11110131011?0011111001100011010010021111 2??0???010?011??21010?0??0???
???1010111111??????1120?1????2?1??2?121211102??05

Massospondylus

10011002100021111111112110111110100001011010001100110111110001000102?01?011
001011010101??1010010010110101010110[01]1010110011100?111002001001111011010002001
11001100000000000000001011101000 1[01]000000000000000?01[01]0111212010111001101111011
12100101211000002000010013100010001101100100110010111000101101000010210101010000
0111011001001111010O?0111110000111110O?01020001100002100 2

Melanorosaurus

1001?1031000211111101?1110101111???101010110101100110101 1?0000001022011011 0
010??200201001101110 1?00111?1000110?10021100110?0101100110110100101111?0000001??00[
12]1000000001000000O?01111001101O0?0011000????0100??22120000011O??????00?0?2100101[

12]0?000102000010013100000101101000 1???1?010110?1110111100111001110111000 00[12]110
1[01]001001101010010011100 1[12]01111200001??101120000110?3

Mussaurus

??
???00100??????10110??0000?1??00100000000?0
0000010??111??0111?00001110?0????0110??101100011110?1?11?00101210110120100000200??
?0?03100010?011??1001?011??10110?10101101000010111101?100000[12]11011101101101010 1
0??????0110111100000112001110000 11003

Neosauropoda

11000113111120110120000010101 11[02]11101112010101011[01]11110121100[01]10112
221110010111120[01]20000010201110000120 1000[01]111111[02]12[01]112[01]00001110011110
10011002012101010 1[01]020111[12]01102012110000210001110111 11[01]1000[01]01001110003?
21001[01]001111000101000000000110001??1?300111101310210001111100110011 10[01]10[01]00
211012??0???010?1111121010?01000011111010111111011??11211100??2?10?20121211102110
5

Neotheropoda

00[01]1[01]002[01]01020100110000 1[01]000000000000011000000000 10[01]100[01]0100000
1[01]0001[012]010000000101100000001110001000000001?00000000000000000101100110000010 0
00[01]100001020000101011001000000010000000000[12]1000011001000000100100001000 10?1100
00010001000120000001 0?0001001100[12]010101013000211101010000111110000110100001?01
10001000000100 0[01]0111111110020010110000011111 0000??0?0000000?010000000000012

Omeisaurus

1100011311112011012000001010 11?20100111201010101111101012110000011222 11100
10111???0?00???1???11100001?0?0001110111112111 3??00111012001101011100 2?121010101 10
20110?011020121100?021???1?10111100100??01111010003221001000 11??????010000100011 00
01001?3101111013101100011111001100011110010021101 2??0???010?010??210100?1?0??111?0
11010?11???1????121?110??2?10?20121211102?105

Ornithischia

0010000000000?0000000?000?1011000100100?1000000100100001000000[01]000100100
00000010000000000010010[01]01[01]000000100111001110100001000000000010100[01]0000??010
000000?00?0?00?000000000000000[12]1000000000000100000100001001 11[02]11000001000000
00000000000000000000000000110112100010001??000?00011000000001000 01?0101111020000?0
00000[01]101110002?01?[01]1000000111100000?000?00?00?0[01]0000000000010

Panphagia

?0????????1??????????0?1???????????????????0??1????????1?1?0???????10?1?111?????
?????????????1?0?0?1010000?10?0101000000???????00000100111?01101?000?01???01?10000
?0?0000000??0????00????0?????00?0?0??000???0?
?0?110002201?00??000????1??0??10???????????????????????00100 1110100???010?1010?0????
????????????0??????????????????

Pantydraco

?0???0????????????[01]00?0???1??1????000100100000010?100001?00????????00?000?1
?10??00100011?000?00?01010??0000?100101010000 1001??11001101010011011 01?00???1????1
????????????????????????0??1?000100000100?????11?01?00???????????????????????????????

????00100121100001?????????0011??10110?00??????????1?1?0000??0000010?01?001????????
????????00001110000 0101?000000001001?

Patagosaurus

11??
?????????????1?00001??????0??0111012111??????11001011000001100 20?2001010?00201?0211
1120121?00?0210001110?01?0?100????0?111?00??2100100?11????????????????????????????1
1110131010000111110011001111 0010?211112??0???0011111???10100[01]??0????????????????
???????????????????????????????????5

Plateosaurus engelhardti

1001100110102011[01]11111211110010110000100101010110011001100011001001 10110
1101101111011000101111001011010111111001010100001100111100110100[01]0110110100020
011100110000000000000001011010000100000000100000000010011111200001100110111101011
100100201000001000010013110010200101000111110010111100001111000010110001010000 11
1111100100110101000010111001011100000010110011000010003

Plateosaurus gracilis

?00??001?0102?110111?1??1110?101??00?10?1010?0??0?10001100??10???????????????
???1?????????????0??0110?????11100101010?001?????1100??01???01101101?00?001?10011000
0000000000010?10100001000000000010000?00????????11200?01100?1011?10101010000???10???
??0?00100131100102001010001111100101 1??0000111100001011 0?0??10000?1??0110?????????
?????????????0??????????????0?0????????[23]

Plateosauravus

??
???10???0100101?01101?00[12]0011?0011000000
0001000000??10???011??00??110??0??????0???[23]01110011100????????????1???????????????
?001001311001 01?01????????11??10110?00001111000010110 00??1000001110110??????0??????
????????????110???0?????0??????????3

Pulanesaura

??
??????????????????????????????10?012011????????001?11?0100??11?????0??1??00[12][01]?001
0?0120001000?????????11111?011???0?0??????????????10??1????????????????????????????????
??????????????????????0?11??1?11????????????????????????000002100101??????????????????
?????????????????????1????????

Riojasaurus

1001?00??0102011?110??0011101101 1000?100001000?10010?0011000100000 1101?0?10
0101?01011??????1?001000?1??101?100010101000010??1111001101001011011010001001 11001
10000000000000001011101?00100000001100 00??0?0100112212100111001111 11?0111110110010
10001020?0010013110010201101000110110010110010110111010110201 10??1000001110110010
011010100???011?0010?1110?0?01?21011[01]00001??13

Ruehleia

??
???10???0????01???101?002001??0011001000001
1000000?11000001?0?00?0110???????0100111012000 11100?0100??01011101000???0??00???00
1001311000020??11000110110?10110?101001010000102 1000??10?00?111011001?000010100??
???????????????????????????????????3

Saturnalia

10?????????????????????????1??1????00?100?0??????????????????10?????1??00???0?0?
?0?0000?????????01000??????0000100000000????????0010010010??01101?0000010000110010
0000000000000001000010???0??1000???0??11101111110001010 2???????????????????????????
?00000111[01]02202100000001001100100100000001001[01]00010110000010000010101000100 10
010100000000100000000000001001 1000?000?0010

Seitaad

??
??00?[01]?1??00??00?001??
??0000????????????????????????????0010011??11000111001 1?11100101310?00?2110??0???0???
???????????0?1000001???????????????????????????????????0?00?11101?0???01101010?00??11?
00??11????0?010???11[01]0000010??

Shunosaurus

11000113?110201101200000001011?20100111201010101001101012000011011121010 00
1011?120020???1102001000001201000111111121211 12??01011?1001?1?10010012?110000101 1
021?0??01??201210000?11??00?101?110110011?111?1?0002221001100?11000?1011001?000110
000??1?310111101310110001111 1?01100?1111001002111 12??0???000?011??21010?[01]1?0????
110?101???1?1011??1121?110??2?00?2?12121?1021105

Silesaurus

00?0??00?0000?00100000??0?00?0?01010?????????0010??0????01?????????00?0?0?000
0??000000?????1?00?0?010???0?0000010001000010?00000000000???0010000000000010100001
100000000000000??100000100000?0?0????????111?11100000100000?????????????????????????
????000001000001001000000000?010100?0?10000110110001001000001000111100000000?00000
00??011?1??002???00000000?0?10???000?0011

Spinophorosaurus

1????1???????????????????????????????????2?????????????0????01?????????0??10111111
0[12]00???????????1?????????????100111012?112?????11?11011?0?01?0002?110??01????[12]?1
1???1?12?1010?0?????????1???11011?001?1??1110?03?2[12]0?10????????????????????????????
??????????????[12]????1?1???01[12]0?011??001??211111110?1101??0?0??20?1002??0??11?101
?0?0?01??????????????????????????????????????

Staurikosaurus

00??
????????????000000001000?00?0000000000????????00010????00?01001?000011?000?0000000
0020000001??010?101100?001100100????1?1??00
0000200110000110?010?00010?00000100001??000?0101?000??000001100000001????01???????
???????????????????????????????????1

Tazoudasaurus

11??010??1?????????[0
2]011???1???1??????????????00?00000???0??10?1111012101???000??001010??????0002??1000
01??00200101011020121000?????????11?11?111?????11?????01?22100100??????????010?2100
00100?0001?310?1???03101??0?111000002??11??1??1??211011110011 11011?0???00100201010
10?1111011111??011????1?????20?????????1??10?1????5

Thecodontosaurus

?0?????????????????????????1??1??1000??????0?0?0?0???000?????????????10?0?0?1010?

?011000???????00?11010??????1100101010000?1??????000101010?1?01101?000001??00110000000000000000?10000?0101000?01000?0?00?01001120110001010011000120100011000010100000101000001210000001???0?000??011??01010?0000111100001011000??10000010101100??011010100?????1100?0?111??0??1??000??00?0????1

Unaysaurus

100110011010??1?111??1??1010?1011000????????????0??00????001???000??0?10??0?10??110100??????010?10110?????01100101010000???0?1?????0?1?0??????????0??0?11?00?10000?0??00000?????????????0?0???010000?00?010011?11200001100??????1????110????211???0??0??110110???011010?00????????0???1100??????0??0???0??????1

Vulcanodon

???[12]????????????????????1??????01?11?0?00??0?0???1???2?[12]10?10?011????????????????????????????????????310[12]?00?01111000100111110010?111011110010011?1????20?10021?0???11?10101101111011??111?10102?1001020111110?121?05

Yunnanosaurus

100?1002??00??10011001??1011111????00101110?00??0?1101111001000001?01?0?00010????????????????10000010???0???00010?21000?1???111100[01]10110100?01101000000110001100000000010000000?0101?10100000?00?0???0??001?0??121200?11100??01??0011?2101100201000[01]020?001001310000000110100010?110010111?00101101000010110101?101000110011001?01101010000111100 1?0?1110?0?011???11?0000110?2
